# Immune Control of Avian Influenza Virus Infection and Its Vaccine Development

**DOI:** 10.3390/vaccines11030593

**Published:** 2023-03-04

**Authors:** Piyush Dey, Akanksha Ahuja, Jaishal Panwar, Poonam Choudhary, Shital Rani, Mandeep Kaur, Akanksha Sharma, Jatinder Kaur, Ashok Kumar Yadav, Vikas Sood, Adukamparai R. Suresh Babu, Sanjay K. Bhadada, Gurpal Singh, Ravi Pratap Barnwal

**Affiliations:** 1 University Institute of Pharmaceutical Sciences, Panjab University, Chandigarh 160014, India; 2 Department of Biophysics, Panjab University, Chandigarh 160014, India; 3 Department of Biochemistry, Jamia Hamdard, New Delhi 110062, India; 4 Department of Chemistry, Anna University, CEG, Guindy Campus, Chennai 600025, India; 5 Department of Endocrinology, Post Graduate Institute of Medical Education & Research (PGIMER), Chandigarh 160012, India

**Keywords:** avian influenza virus, innate immunity, adaptive immunity, diagnosis, vaccine development

## Abstract

The avian influenza A virus (AIV) is naturally prevalent in aquatic birds, infecting different avian species and transmitting from birds to humans. Both AIVs, the H5N1 and H7N9 viruses, have the potential to infect humans, causing an acute influenza disease syndrome in humans, and are a possible pandemic threat. AIV H5N1 is highly pathogenic, whereas AIV H7N9 has comparatively low pathogenicity. A clear insight into the disease pathogenesis is significant to understand the host’s immunological response, which in turn facilitates the design of the control and prevention strategies. In this review, we aim to provide comprehensive details on the pathogenesis and clinical features of the disease. Moreover, the innate and adaptive immunological responses to AIV and the recent studies conducted on the CD8^+^ T cell immunity against AIVs are detailed upon. Further, the current status and advancement in the development of AIV vaccines, along with the challenges, are also discussed. The information provided will be helpful in combating the transmission of AIV from birds to humans and, thus, preventing severe outbreaks leading to pandemics worldwide.

## 1. Introduction

Influenza viruses (IVs) of types A and B lead to seasonal influenza epidemics, but only type A is linked to pandemics. The two main viral surface proteins that cause protective host antibody responses are haemagglutinin (HA) and neuraminidase (NA), which are categorized into 18 HA and 11 NA subtypes based on an antigenic investigation. The IV is an eight-segmented single-stranded RNA virus. Mutations cause genetic and antigenic variations in RNA viruses, just as in other viruses. The virus’s segmented RNA genome allows it to produce a variation through genetic reassortment. The predominant human H1N1 IV acquired a novel HA and polymerase basic 1 (PB1) gene of avian origin, resulting in a virus with a new subtype: H2N2 in 1957 and H3N2 in 1968. This led to the pandemics of 1957 and 1968 [1]. AIVs belong to the category of type A viruses because they can infect humans and animals. Type A viruses are a significant danger to public health, as they can cause an influenza pandemic. AIVs are carried by wild birds in their intestines. Thus, the birds shed these viruses in saliva, feces and nasal secretions. AIVs are found naturally in aquatic birds. Except for H17N10 and H18N11, which are found in bats, all the subtypes are prevalent in aquatic birds [2]. AIVs are classified according to their potential to cause disease in chickens. AIVs can be low pathogenic AIVs (LPAIV) or high pathogenic AIVs (HPAIV). LPAIVs only cause mild illness in domestic birds, whereas acute influenza symptoms are manifested in the case of HPAIVs, which may also cause internal organ damage, affect the respiratory tract and increase poultry mortalities [3]. LPAIV is the main cause of upper respiratory tract (URT) infections with variable degrees of sinus, conjunctival and tracheal irritation. Influenza H5 and H7 subtypes arise from non-pathogenic influenza viruses belonging to the category of HPAIVs [2]. H5N1, H7N2, H7N3, H7N7, H9N2, H7N9, H5N6, H6N1, H10N7 and H10N8 are AIVs known to infect humans. [2,4]. Only mild clinical symptoms are observed in infections with H7N2, H7N3, H7N7, and H6N1 [4]. However, some AIVs, such as H5N1 and H7N9, can cause severe influenza disease and have high human mortality rates [5]. The reason behind this is the truancy of preexisting AIV-specific antibodies, although antibodies against both viruses can be identified during the development of illness [2].

The H5N1 AIV continues to spread zoonotically among people, causing severe illness and posing a pandemic threat [6], and Early in 2009, a novel H1N1 variety (H1N1v) virus of a swine origin initially surfaced; it is currently a pandemic [7]. This contradicted the conventional belief that an influenza pandemic is brought on by the advent of a virus with a unique HA subtype. Except for those over 60 years old, there is minimal preexisting cross-reacting humoral immunity in the community, and the new H1N1 virus is antigenically distinct from the prevalent human H1N1 virus [8]. As a result, the human population is already endemic to a universal strain of the influenza subtype H1N1. To put an end to the COVID-19 crisis, several mitigation measures, including face masks, social distancing and even quarantine, have been put into place. Despite this, the possibility of a subsequent human pandemic exists due to the emergence of AIV during COVID-19 [9]. The introduction of AIVs into the naïve population may lead to devastating consequences. However, the virus must circumvent the person-to-person transmission hurdle to become a pandemic after trouncing the barrier between animals and humans. Over the last hundred years, only three AIV subtypes, A/H1, A/H2 and A/H3, gained the potential for transmission between humans. This transmission ensued in the Spanish pandemic (A/H1N1) in 1918, the Asian pandemic (A/H2N2) in 1957, the China pandemic (A/H3N2) in 1968, and the A/H1N1pandemic in 2009 [10]. The expanding emergence of zoonotic AIVs, and the mortality associated with influenza A viruses emphasizes the critical need for the world to be prepared for these pandemics. Initially, it was thought that AIVs could only spread in humans. However, after the initial human cases of the avian H5N1, an increase in the cases of zoonotic incidents appearing from chickens has been reported. Since then, the AIVs’ subtypes A/H5, A/H6, A/H7, A/H9 and A/H10 have also been identified as causing infections in chickens [10]. There is variability in the intensity of the illness caused by different viruses, which is distinguished by symptoms such as encephalitis, conjunctivitis, symptoms such as influenza, and pneumonia linked with the acute respiratory distress syndrome (ARDS). Among all the AIVs, H5N1 and H7N9 are of great concern, as they stand out because of their rate and number of fatalities. The avian influenza A (H5N1) strain can induce inflammatory and cytokine responses in both humans and birds with high mortality rates [11]. H7N9 is a potential influenza pandemic virus as: (i) It is widespread in the poultry market and overcomes host barriers, (ii) it has a reassortment with the local H9N2 and easily becomes adapted to a human host and (iii) at the population level, preexisting neutralizing antibodies are absent. 

Genetic reassortment of AIVs has resulted in the occurrence of three pandemic IVs: H2N2, H3N2, and H1N1, which are known to impact the viral infectivity and ability of transmittance to mammals [12,13,14]. The antigenic drift and antigenic shift are two processes by which IVs undergo antigenic modification. Antigenic drift occurs when small mutations in the HA or NA glycoproteins arises due to the host immunity’s selection influence [15]. Antigenic shift occurs when the eight gene segments undergo genetic reassortment to create new HA/NA subtypes, which the general populace of humans has significantly less to no immunological defense against [16]. Understanding how IVs interact with their hosts and how environmental and societal variables affect these interactions is vital in order to respond to the newly developing and reemerging influenza illnesses [17]. Studies on the interactions between hosts and pathogens can assist in understanding the molecular pathogenesis of the disease and in developing the effective preventative and treatment measures against influenza [18,19]. 

When an infection is present, the host’s innate immunity acts as the first line of defense and produces pro-inflammatory reactions [20]. Adaptive immunity also plays an essential role in removing viral pathogens during the later stages of infection. Furthermore, during IAV infection, the respiratory mucosal immunity is generated in the associated mucosal tissues and is engaged in an antiviral defense. IAVs have evolved a variety of ways to escape the host defense and can establish an effective infection, despite the presence of several immunological systems to destroy the invading pathogens or to limit viral replication [21]. Therefore, it is crucial to comprehend how the human immune system reacts to AIVs to effectively manage severe H5N1 or H7N9 influenza illness and logically develop novel immunotherapies and vaccines. Understanding the pathogenesis and immunity of AIVs in humans is very important, especially for those strains that overcome the host barrier to infect people [2].

This review highlights the most recent findings on the epidemiology of AIVs, clinical signs and symptoms, and immunological responses to AIVs, including innate, cellular and humoral immunity to AIVs. The essential role of the immune responses that assist the host recovery from AIV infections in critically ill patients has also been highlighted, which might prove helpful in formulating novel immunomodulatory interventions against severe AIV infections. Further, critical diagnostic strategies for detecting AIVs have been detailed. In addition, the critical role of vaccine development and the various underlying challenges to vaccine formulation has also been explicated in this manuscript.

## 2. Epidemiology of Avian Influenza Viruses 

IVs are a significant menace to public health and can cause mild to severe respiratory infections in humans. According to the World Health Organization (WHO), seasonal IVs, including H1N1 and H3N2 IAVs and influenza B viruses, cause around 3–5 million severe cases and 290,000–650,000 fatalities globally each year [22]. Additionally, AIVs such as H5N1, H7N9, and others can lead to many zoonotic infections. Pandemics are triggered when viruses from the animal pool transcend the species hurdle, generally because of an antigenic change during a reassortment stage between an AIV and a human IV. These pandemics can kill millions of people and cause more complications and mortalities than seasonal IV outbreaks [23]. Four hundred and forty years have passed since the “Spanish Influenza” pandemic. During this severe pandemic of 1918, about 500 million (about one-third of the world population) contracted the virus, and between fifty to hundred million people were reported to be killed [24]. Avian H1N1 viruses entered the swine population of Eurasia and were reported to be co-circulating with classical swine flu viruses in 1979 [25]. An H1N1 strain that swept through India in 2015 resulted in around 10,000 incidents and 774 mortalities [24]. Most of the time, pandemics are brought on by viruses with surface glycoproteins such as HA and NA, which, in dealing with, the human immune defense system is not very efficient. This was true in 1918, when most people appeared to be unaware of both the H1 HA and the N1 NA, and in 1957, when people were essentially resistant to both the H2 and N2 viruses. Only the H3 HA was transmitted to people for the first time in 1968, although the N2 of the H3N2 pandemic virus was obtained from the formerly perpetuating H2N2 virus. In 2009, a seasonal strain of the H1N1 virus infected people, but the pandemic strain contained antigenically different H1 and N1 surface glycoproteins [26]. The H3N2 virus became widespread among humans during the 1968 pandemic, and it has been the cause of numerous influenza pandemics ever since. H3N2 viruses have arisen from various sources, including domestic poultry, pigs and wild birds. Dogs have developed severe respiratory illnesses because of some H3N2 viruses that originated in birds. If different lineages of the H3N2 virus were passed on to humans, there would be a possibility of a human influenza pandemic [27].

**Figure 1:**
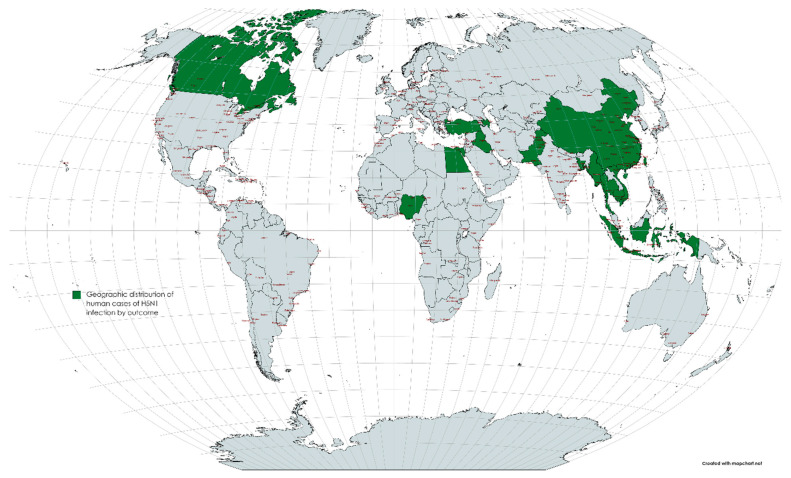
Geographic distribution of human cases of H5N1 infection by outcome, between May, 1997 to April, 2015.

**Figure 2:**
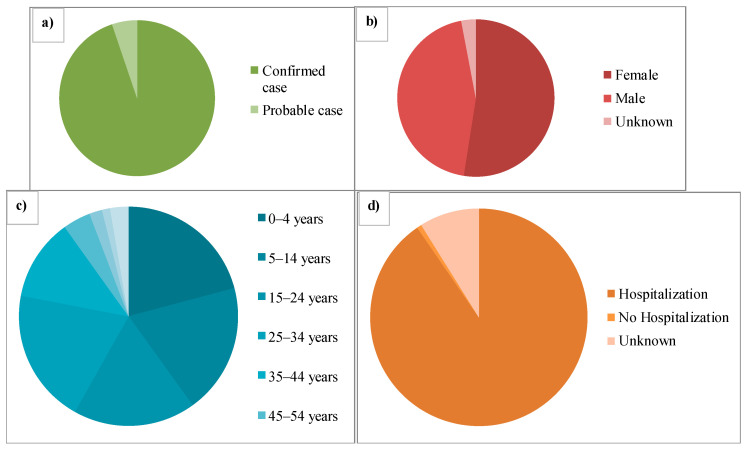
Demographic and epidemiologic characteristics of human cases with H5N1 virus infection by outcomes, between May 1997–April 2015. (**a**) Represents relative percentage of human cases based on types of cases, (**b**) shows relative percentage of human cases based on sex, (**c**) shows relative percentage of human cases based on age and (**d**) represents relative percentage of human cases based on hospitalization.

The first infection due to the highly pathogenic H5N1 subtype was described in China in 1997, and the virus was found out to be of the A/goose/Guangdong/1/1996 lineage (GsGd), which caused 6 fatalities and 18 human infections. Viruses from this lineage caused a human infection again in 2003 [28]. For H5N1 virus infections, there is a human incubation period of 2 to 5 days, and varies up to 17 days [29]. A combined 907 human H5N1 cases were described worldwide between May 1, 1997 and April 30, 2015, of which 94.8% were established cases and 5.2% were possible cases. A total of 16 countries reported human instances between 1997 and 2015. As the illness migrated from East Asia to Southeast Asia, then West Asia, North Africa and other regions, more countries became afflicted between 2003 and 2008. (Figure 1) Cases are also reported almost annually in China, Vietnam, Cambodia, Indonesia and Egypt. While the incidence in Asia stayed low from 2013 to 2015, the number of cases in Egypt increased in 2014 and 2015. In addition, 67.2% of the cases recorded between December and March between 1997 and 2015 peaked in January. The geographic dissemination of the H5N1 illness in humans by outcome between May 1997 and April 2015 is depicted in green on the world map in Figure 1. From 1997 to 2014, the general male-to-female proportion was about equal (1:1.2), albeit this trend varied by location. The average age of the cases was 19, and the interquartile range (IQR) ranged from 5–32 years. Of all the cases, 41.2% belonged to children under the age of 15, and 80.3% of the involved individuals fell under the age of 35 [30,31,32]. (Figure 2) The average age of the cases varied depending on their result, with fatal cases having an average age of 22 years and recoveries having an average age of 10 years. Table 1 illustrates the major AIV outbreaks in the past 30 years, along with the significant clinical symptoms reported in the case of the respective outbreaks.

The first human infection attributed to the novel H5N6 virus was identified in China, in April 2014. The outbreak of H5N8 viruses occurred in 2014 among wild birds and several poultries across the globe. In early 2014, many outbreaks amongst domestic ducks and migratory birds were reported in South Korea. As a result of these outbreaks, two subtypes of H5N8 were identified: Buran 2-like and Cochang 1-like. Since then, several episodes of these viruses have been reported in numerous domestic poultries across different continents [33]. In December 2020, cases of human infection by the H5N8 influenza virus were reported. Seven poultry workers contracted the virus; they were between 29 and 60 years of age, and included two males and five females [34]. 

The H6 subtype of AIV was first isolated in US in 1965 from a turkey. H6 AIVs were able to infect ducks and chickens, and they circulated in the US’s live poultry market. The H6N1 virus was found in dogs in China in 2014. Its molecular analysis revealed that it is closely related to the H6N1 virus in humans [35].

Viruses belonging to the H7 subtype can infect a broad range of species, including wild birds, mammals, seals, pigs, horses and humans. H7 is an LPAIV, which circulates asymptomatically in poultry but evolves as HPAIV, and can cause systemic diseases or even mortality. Humans have been reported to get infected sporadically from poultry. One death during the outbreak of HPAIV H7N7 was reported in the Netherlands in 2003. Three instances of mild H7N7 infections in humans were discovered in 2013 in Italy while observing the employees from the latest H7N7 epidemic at a poultry holding. H7N9 is an LPAIV that emerged in humans and poultry in China in February 2013. This virus also contributed to seasonal waves of zoonotic infections in China. [36]. Majority of human infections originated due to contact with poultry and not because of person-to-person transmission [37]. Human illnesses caused by H7N9 have been more severe since 2013 than all the prior H7 outbreaks in humans [38]. Since March 2013, the A/H7N9 virus has infected 657 individuals with a 38% fatality rate after crossing the host barrier from birds to humans [39]. This is unexpected, especially when considering the 1918 Spanish influenza pandemic, which was thought to be exceedingly severe and had a mortality rate of more than 2.5% [40,41,42]. More than 20 million birds were killed in Mexico in 2013 as a consequence of an HPAIV H7N3 virus pandemic and two verified minor human infections. H7N3 viruses have been found in poultry in some countries, including Chile and Canada [43,44,45]. 

In 1966, the first H9N2 viruses were isolated from turkeys in Wisconsin. This virus has been isolated from domestic ducks and wild birds throughout Eurasia and occasionally from poultry during sporadic outbreaks in the northern United States [46]. With the quick differentiation of H9N2 AIVs, more than 102 genotypic variations have been identified using the nomenclature scheme [47]. The ubiquity and elevated frequency of the mutation of H9N2 IVs, along with their capability to transmit internal genes to other AIVs and adjustment to the human host, may be key early indicators of the occurrence of new reassortants with pandemic capabilities.

H10 AIVs have been isolated from a variety of mammalian and avian species. There have been sporadic reports of influenza infections due to this subtype, although person-to-person transmittance has not been proven. Two infants in Egypt in 2004 and two abattoir workers in Australia in 2010 were found to be infected with the H10 virus during an outbreak of avian influenza among chickens in Australia. It evolved from the internal genes of enzootic H9N2 viruses in chickens and H10 and N8 viruses’ surface genes from household ducks. This resulted in three deadly diseases and two fatalities in people [48,49,50,51,52,53,54,55,56,57,58,59,60]. H10 AIVs have also been detected in mink, seals, mammals and pigs [61,62,63,64,65,66,67,68]. 

**Table 1:** Major avian influenza virus outbreaks that occurred in the past 30 years.

Influenza Strain	Year	Location	Clinical Symptoms	References
H5N1	1997 2003 2008 2003–2017 2015–2019	China China India Thailand, Indonesia, China, Vietnam, Azerbaijan, Cambodia, Iraq, Egypt, Turkey, Laos, Nigeria, Myanmar, Canada, Bangladesh, Djibouti, Laos Egypt	Flu-like symptoms Pneumonia Influenza-like illness Pneumonia Influenza-like illness	[2,29] [52,53] [54] [53] [55]
H5N2	2015	United States	Neurological signs, lethargy	[56]
H5N8	2014 2021 2020	China China Russia	Fever Headache, nasal stiffness, cough, fever Asymptomatic	[57] [58] [59,60]
H6N1	2013	China	Cough, fever, muscle ache, headache	[35,61]
H7N2	2002 2007 2016–2017	Virginia UK USA	Influenza-like illness Conjunctivitis, influenza-like illness Conjunctivitis, sore throat, muscle aches, cough	[45] [38] [62]
H7N3	2004 2006 2012	Canada UK Mexico	Conjunctivitis Conjunctivitis Conjunctivitis	[38]
H7N7	1996 2013 2015	UK Italy Netherlands	Conjunctivitis Conjunctivitis Conjunctivitis, mild influenza-like illness	[38] [38] [38,63]
H7N9	2013	China	Pneumonia-like symptoms	[29,36]
H9N2	1998 1999 2003 2011 2021	China China China Bangladesh Cambodia	Symptoms similar to flu Symptoms similar to flu Symptoms similar to flu Symptoms similar to flu Influenza-like illness	[64] [64] [64] [65] [66,67]
H10N7	2004 2010	Egypt Australia	Cough and fever Conjunctivitis	[51] [2,68]
H10N8	2013	China	Pneumonia	[2,51]

## 3. Pathogenesis and Clinical Features of Severe Disease

Because the human population is immunologically unprepared for the aggressive HA subtypes, there is a small but significant danger of human-to-human transmission of the AIVs, which might result in a devastating pandemic [69,70]. An efficient reaction to the H5N1 AI pandemic or any other appearing and re-appearing disease necessitates a thorough interdisciplinary strategy for preparedness [17]. Understanding the relationship between the host and the infection might be helpful in developing better influenza vaccines and antiviral medications. Such investigations will, from the perspective of comparative biology, demonstrate the comparative molecular pathophysiology of the influenza illness and how healthy cellular functions operate [71]. 

### 3.1. Pathogenesis of AIV Illness in Gallinaceous Birds

**Figure 3:**
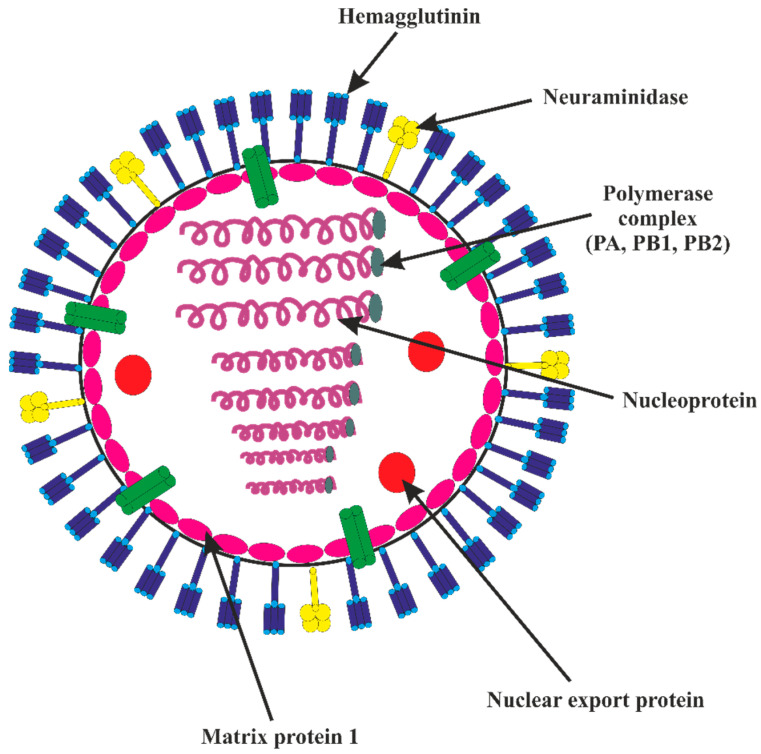
Schematic diagram of avian influenza virus illustrating its various components. The segmented RNA genome of influenza A virus encodes three proteins that make up the virus’s envelope—hemagglutinin (HA), neuraminidase (NA) and the ion channel M2 protein—as well as internal nucleoprotein (NP), polymerases (PA, PB1 and PB2), matrix protein 1 (M1) and non-structural proteins, which are all encoded by the virus (NS). The lipid bilayer of the virus is generated from the host cell membrane.

**Figure 4:**
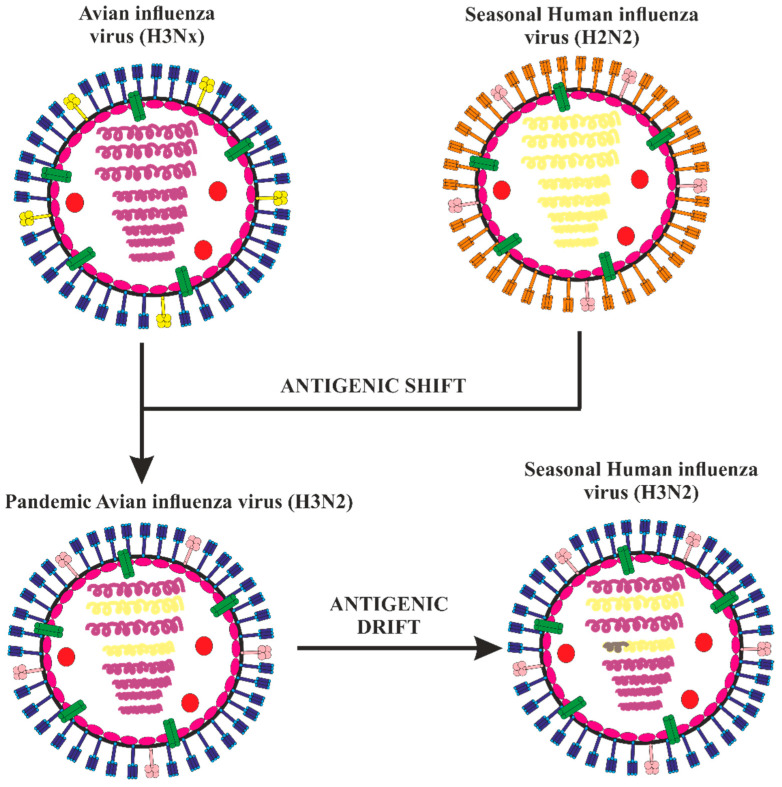
The seasonal human influenza virus and an avian influenza A H3Nx (where x = 1–9) virus co-infected each other in 1968. The RNA polymerase PB1 and hemagglutinin (HA) RNA segments of the pandemic human influenza A H3N2 virus were selected as a result of the reassortment of viral segments between A H2N2 viruses, with the other segments being taken from the human virus. As with all other human seasonal influenza viruses, once H3N2 had established itself in people, it started to drift. Although not as significantly as during shift, modest antigenic alterations in the HA protein caused by mutation were chosen to promote immune evasion during drift. Figure is adapted from Krammer, 2018 [108].

**Figure 5:**
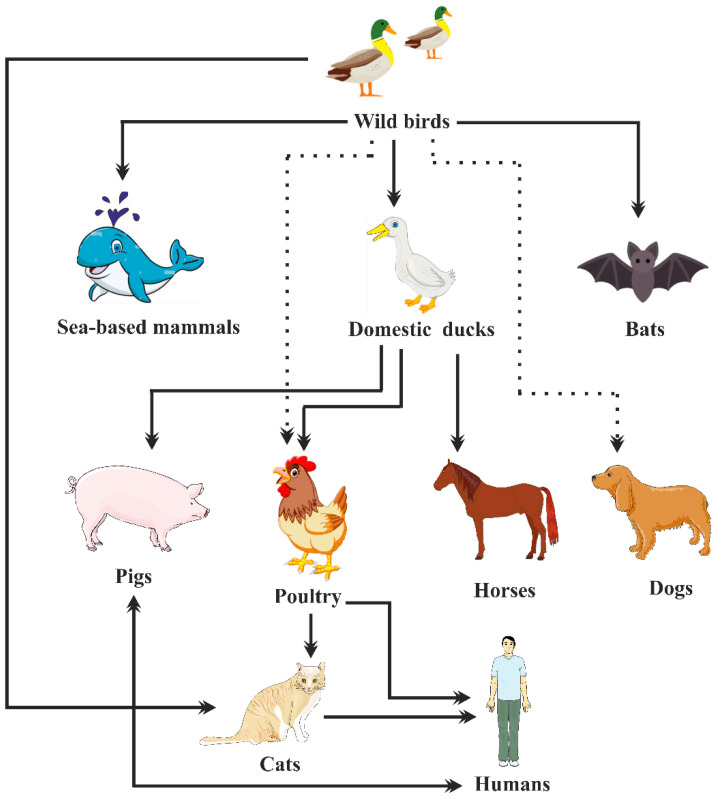
Emergence of influenza A virus from reservoirs of wild aquatic birds. Wild bird influenza viruses can spread to marine animals and domestic free-range ducks via water or fomites. Transmissions to other avian species (such as poultry) can result from contaminated water, domestic ducks or directly from wild birds. Dogs and cats are other household animals that are susceptible to influenza virus infections. Influenza virus can also be transmitted from poultry to humans, who can also acquire it from cats. Transmission that avoids a domestic duck intermediary is represented by dashed lines.

HA is a critical factor in avian virulence, but the best internal gene combinations are necessary for maximal virulence expression [72]. The structure of an AIV is illustrated in Figure 3, while a schematic diagram showing antigenic drift and antigenic shift is depicted in Figure 4. The emergence of IAV from reservoirs of wild aquatic birds is pictorially depicted in Figure 5. For an infection to begin in birds, HA must attach to -2,3-galactose linkage cell receptors and prompt receptor-regulated endocytosis. This influences host particularity and cell or tissue reaction. As receptor-binding alterations occur, the variety of hosts that IVs can infect may also vary [73,74,75,76]. The distribution of these viruses in the tissues is determined by this breakdown pattern [74,77]. However, after an intranasal infection, H7N1 LPAIV RNA was found in organs other than the respiratory and digestive systems, demonstrating that the virus travels systemically [78]. Within 16 h of intranasal contact, the HPAIVs multiply in nasal epithelial cells. Within 24 h, they reach visceral organs, and by 48 h, the virus titers may shoot up to the maximum, leading to severe lesions in several visceral organs [79]. The capacity of HPAIVs to reproduce in macrophages, heterophils, and endothelial cells is crucial for the virus’ ability to move to other organs via the lymphatic and circulatory systems, where it multiplies in parenchymal cells [79,80,81]. Additional details on pathogenesis of the virus illness have been succinctly discussed in Supplementary Materials, Section S2.

### 3.2. Pathogenesis of AIV Illness in Non-Gallinaceous Birds

The current Eurasian-origin HPAI H5N1 viruses connected to the Gs/Gd virus target many hosts [82]. Additionally, they can cause mild to severe illnesses in non-gallinaceous birds. Several aquatic and terrestrial wild bird species have been observed to perish since 2002 as a result of the Eurasian-African H5N1 HPAIV infection [83,84,85,86]. Ten separate genetic lineages (clades 0 to 9) and various sub-lineages (e.g., 2.1.1, 2.1.2, 2.3.4.4, and others) have emerged in the H5N1 HPAIVs of the Eurasian-African lineage. For a range of bird species, these various influenza viruses have demonstrated distinct clinical traits and enhanced pathogenicity [79]. For instance, the HPAI H5N8 virus (clade 2.3.4.6) kills a lot of Baikal teals, quails, and bean geese; however, it has no symptoms in mallard ducks [85,87,88]. Additionally, these H5 HPAI lineages reproduce in more significant titers, which help the virus spread to the vulnerable wild duck population [85,87,88]. A similar viral pathogenesis occurs in both ducks and chickens, except for ducks, which have neither viral replication in the endothelium nor any accompanying endothelium lesions [89]. 

### 3.3. Pathogenesis of AIV (H5N1) Infection in People

**Figure 6:**
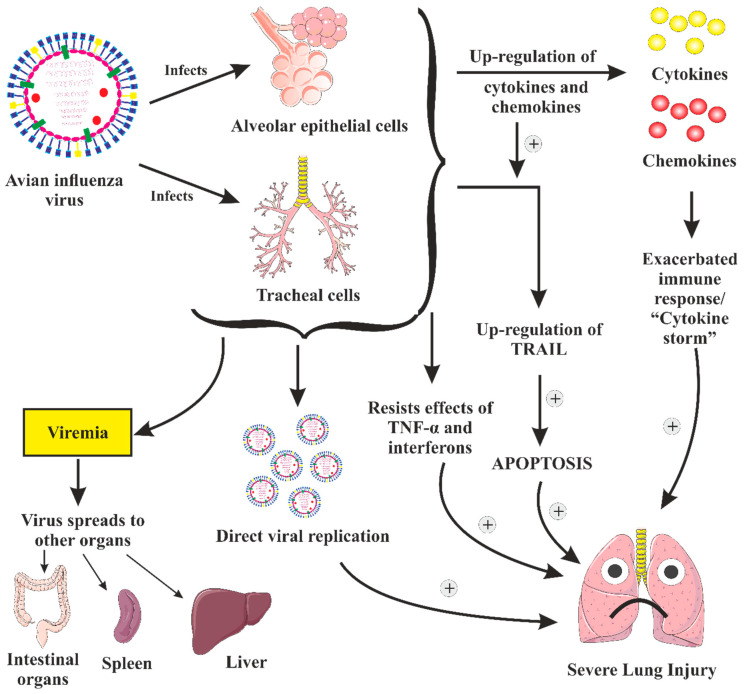
Pathogenesis of H5N1 virus infection. Virus infects alveolar and tracheal cells, and induces up-regulation of pro-inflammatory cytokines, leading to an aberrant and exacerbated immune response/”Cytokine storm”, which contributes to induction of severe lung injury; stimulation of cytokines and chemokines also activates TRAIL, which leads to apoptosis of infected cells and macrophages, thus leading to lung damage; direct viral replication in the body also contributes to induction of severe lung injury; infection of alveolar and tracheal cells prompts the dissemination of infection to immune cells (viremia), which leads to spread of infection to other organs of the body such as intestinal organs, spleen and liver.

If the H5N1 virus mutagenically transforms into a form that transmits amidst people, a pandemic of the highly deadly infectious illness H5N1 avian influenza may ensue. The lungs of infected individuals usually experience diffuse alveolar destruction, bleeding, and infection, specifically in isolated lung epithelial cells. The virus might additionally affect other organs such as the trachea, intestines, and brain [90], in addition to have the ability to pass across the placental hurdle and affecting the fetus (Figure 6) [91]. In addition to the viral replication in humans, the irregular modulation of cytokines and chemokines might have a significant function in the pathogenesis of the H5N1 influenza. Other factors, including elevated levels of the apoptosis-inducing ligand, TNF-related apoptosis-inducing ligand (TRAIL) and lower cytotoxicity of CD8^+^ cells, are also suspected to be engaged. However, it is still unclear how exactly they lead to the infectivity of the H5N1 influenza.

Numerous studies suggest that the pathophysiology of the H5N1 influenza may be significantly influenced by the abnormal production of proinflammatory cytokines and chemokines. In H5N1 autopsy cases, pathological characteristics that are associated with cytokine and chemokine dysregulation, such as hemophagocytic activity, have been observed [92,93,94]. Proinflammatory cytokines and chemokines have been found in significant amounts in the serum of several H5N1 patients [92,95,96]. The results of in vitro tests also confirm that an exacerbated immune response plays a part in the infectivity of the H5N1 influenza. In comparison to human influenza viruses, H5N1 avian influenza viruses dramatically increase the production of several cytokines and chemokines in human macrophages and respiratory epithelial cells [97,98,99]. The increased cytokine and chemokine production in the supernatants from the infected cells indicates the increased expression in these tests. In addition to their potential to upregulate cytokines and chemokines, H5N1 viruses may also be able to resist the antiviral effects of interferons and TNF-α [100] (Figure 6).

Stimulating a functional TRAIL in H5N1 influenza virus-affected macrophages may play a substantial role in the pathogenesis of this illness (Figure 6). TRAIL is one of the several death receptor ligands that bind to the receptors for death receptor ligands produced on target cells, inducing cell apoptosis (Figure 6) [101]. In Zhou and colleagues’ study, the TNF and TRAIL expression was demonstrated to be much higher in macrophages affected with the H5N1 IV in vitro compared to macrophages affected with the human H1N1 IV [101]. Furthermore, it has also been demonstrated that virus-infected T cells are more sensitive to the ligand-induced apoptosis. Both TRAIL sensitization and up-regulation may partially explain the lymphopenia and lung damage typically observed in H5N1 patients. Additionally, compared to H1N1-infected macrophages, it has been described in vitro that H5N1-infected macrophages exhibit a delayed beginning of apoptosis [102]. The longer lifespan of the affected macrophages further stimulates the apoptosis of T cells. The extended duration of the cytokine and chemokine production by macrophages may contribute to an increase in the immune-mediated illness. Human autopsies have revealed that leukocytes and alveolar epithelial cells all experience apoptosis in the lungs, spleen and intestinal organs [103]. Therefore, apoptosis could be one of the pathogenic pathways causing damage to the lungs and other organs. Apoptosis may be brought on by direct viral multiplication and the stimulation of the production of cytokines and chemokines.

In humans and mice, a severe infection with avian IAVs is characterized by significant lymphopenia [104,105]. In one of the studies, it was demonstrated that the mice infected with the lethal H5N1 strain had higher amounts of plasmacytoid DCs (pDCs) expressing the Fas ligand (FasL), which caused the death of CD8^+^ T cells specific for influenza via a Fas-FasL-mediated pathway; the mice inoculated with the non-lethal H5N1 strain did not exhibit this [106]. Furthermore, it was demonstrated that mice infected with the lethal H5N1 virus accumulated more pDCs than other DC subsets in the lymph nodes (LNs) that drain into the lungs, and that the rise in FasL expression on pDCs was accompanied by elevated levels of the IL-12p40 monomer/homodimer in these LNs. The findings implied that pDCs exhibited a negative function in one of the lymphopenia-related pathways of the deadly H5N1 virus infection.

In vitro findings also demonstrate that the HAs of H5N1 viruses decrease perforin activity in cytotoxic T cells, in contrast to H1N1 and H3N2 viruses [107,108]. This may result in a diminished cytotoxic function preventing the clearance of cells, such as the antigen-presenting cells (APCs) that carry the H5N1 virus or the HA (H5) protein. The chronic antigenic stimulation of cytotoxic T cells that causes excessive IFN production may also stimulate macrophages to produce proinflammatory cytokines more aggressively, which might further amplify the immune response in influenza patients.

### 3.4. Clinical Findings of H5N1 Infection

H5N1, which causes a variety of ailments, including severe and deadly respiratory conditions, can be challenging to diagnose. Despite the prevalence of some less severe illnesses, the hospitalization of patients with severe diseases has also been reported, aggravated by ARDS and multi-organ failure [30]. High viral loads, lymphopenia, exceptionally elevated circulatory levels of the IFN-inducible protein-10 (IP-10) and elevated levels of inflammatory cytokines and chemokines have all been linked to fatal outcomes in H5N1-infected patients [109,110]. Hemophagocytosis has also been observed in patients with a severe H5N1 infection [111]. Sputum production can occasionally be bloody, and it fluctuates in quantity. Radiographic changes include segmental or lobular consolidation with air bronchograms; diffuse, multifocal, or patchy infiltrates; and clinically apparent pneumonia in almost all instances [112]. Multi-organ failure has frequently been noted, together with symptoms of kidney malfunction and occasionally cardiac dilatation and supraventricular tachyarrhythmia [112,113,114]. 

In 1997, China had the foremost human epidemic of the avian influenza A (H5N1) virus. Six people who had the infection were proven dead. Without a middle host, infections were directly transferred from chickens to people. Asymptomatic illnesses to deadly pneumonitis and multiple organ failure are all within the clinical range of H5N1 infection. The most common pathologic finding was a reactive hemophagocytic syndrome, which may have led to lymphopenia, liver failure and aberrant clotting. The epidemic management benefited greatly from a quick diagnosis, which was made possible by a reverse-transcription polymerase chain reaction (RT-PCR) and a monoclonal antibody-based immunofluorescent test [113]. 

As per the examination of the case notes of 12 individuals with an H5N1 infection, confirmed by viral culture by an investigation in February 1998, seven individuals had a pneumonia-like disease with a clinical presentation resembling the flu [115]. Pancytopenia, elevated liver enzymes, and gastrointestinal symptoms were notably evident. Furthermore, for the quick identification of viruses in respiratory specimens, an H5-specific RT-PCR test proved effective. A widely available enzyme immunoassay for quick viral diagnosis proved more accurate than direct immunofluorescence. In addition, for the immediate exclusion of H5-subtype illness, direct immunofluorescence using a pool of monoclonal antibodies specific to H5 also proved effective. 

### 3.5. Clinical Findings in H7N9 Infection

Rapidly progressing pneumonia brought on by H7N9 infection in humans is accompanied by leukopenia, lymphopenia and significantly elevated blood cytokine and chemokine concentrations [45]. In a case study of 111 patients, 97.3% of patients with avian H7N9 infections had pneumonia and 88.3% had lymphopenia, and in other cases in a series of 216 patients, it was reported that 61.3% of patients were taken to intensive care [5,116,117]. During the severe phase of infection, neutrophil-related immunity and the cell cycle was activated, while T cell processes were stimulated during the recovery period. In eight patients, the transcriptional profiling revealed the similar results, while mechanical ventilation or extracorporeal membrane oxidation was required for each of them [118].

During seasonal influenza epidemics, hospitalization rates for older adults were higher [48]. Children and young people spend more time in hospitals [2]. Males older than 60 demonstrated signs of a more severe illness during the initial wave of H7N9 infections [119]. Aging causes immunosenescence in the innate and adaptive immunological systems, which reduces the body’s ability to respond to the influenza infection and vaccination [120]. Some of the clinical and pathological characteristics of the severe A/H7N9 sickness brought on by the AIV infections included leukopenia, lymphopenia, viral spread in extra-pulmonary locations, extended viral discharge of H7N9 viral RNA in feces and urine, and multiple organ failure [121,122]. Indeed, in patients with H5N1 infection, large virus levels were also linked to death [123], while a reduced viral load during the latest H7N9 epidemic was linked to recuperation [2]. Pneumonia and ARDS are the frequent causes of AIV fatalities [123]. The hospitalized patients exhibit symptoms of viral pneumonia and bacterial co-infections [124]. However, patients in hospitals receive broad-spectrum antibiotic therapy [125], which makes detecting bacterial co-infection difficult. Type I/II diabetes and hypertension were prevalent among the hospitalized reports of illness due to the H7N9 influenza [126]. Interestingly, young infants with confirmed instances of H5N1 and H7N9 infections showed mild influenza symptoms [127]. Clinically, the children had a fever, but no ARS-related signs, such as pulmonary edema, were observed, which more specifically includes aging-related alterations to the immune system. 

### 3.6. Clinical Findings in H5N6 Infection

The first severe avian influenza infection in humans with an H5N6 virus was found in China in 2014. The virological and clinical effects of a fatal H5N6 virus infection in a human patient were demonstrated in a 2015 investigation. The patient, who had a fever, acute pneumonia and lymphopenia at the beginning of the illness, went into septic shock and had ARDS, and succumbed on the 10th day of the disease [128]. The patient’s trachea or throat swab was used to isolate a unique reassortant H5N6 virus. Multiple basic amino acids were present at the cleavage location of the HA gene, demonstrating the high infectivity of the new H5N6 virus in chickens. Recently, an investigation also presented the first severe H5N6 virus-induced case of acute encephalitis with moderate pneumonia. A 6-year-old girl was taken to the hospital on January 25, 2022 with severe neurological symptoms, which quickly progressed to seizures and coma. Imaging of the brain revealed anomalies. Laboratory tests demonstrated that the serum’s transaminases, lactate dehydrogenase, and cytokines were unusually increased. From the patient’s blood, CSF, and tracheal aspirate samples, a unique reassortant H5N6 virus was found. According to a phylogenetic study, this virus, which belonged to the clade 2.3.4.4b, was a unique reassortant influenza A H5N6 virus of an avian origin. An epidemiological examination established that the direct source of the virus, in this instance, was wild ducks [129]. 

### 3.7. Clinical Findings in H7N2 Infection

An immunocompromised male with a fever and community-acquired pneumonia was found to be infected with a low pathogenic avian influenza A (H7N2) virus in New York, NY, USA, in 2003. The patient’s early complaints were related to ophthalmological symptoms. The fact that the examinations were carried out five months after the patient was admitted to the hospital, after which the influenza A isolate was recognized as an LPAI A H7N2 virus, was a study restriction. In addition, the patient’s medical history and clinical results were in accordant with an HIV infection and the community-acquired pneumonia, with a potential improvement from viral pneumonia or a clinical response to the antimicrobial medication therapy [130].

### 3.8. Clinical Findings in H7N7 Infection

A case of an HPAI H7N7 virus subtype first appeared in widespread poultry farms in the Netherlands around the end of February 2003. An epidemic investigation was initiated to determine the degree of the IAV subtype H7N7 transmission from chickens to people, even though the danger of transmission to humans was previously believed to be minimal. A total of 453 persons reported having health issues, including 90 cases of influenza-like sickness, 349 reports of conjunctivitis and 6 other complaints. The study found A/H7 in conjunctival samples from seventy-eight individuals who only had conjunctivitis, five individuals who also had influenza-like illness and conjunctivitis, two individuals who just had the influenza-like disease, and four individuals who reported additional symptoms. Out of eighty-three contacts screened, three had the A/H7 infection, and one became sick with the flu. Six people were infected with influenza A/H3N2. All employees who encountered the diseased persons were mandated to undergo the influenza virus vaccination and oseltamivir preventive therapy after 19 persons had been diagnosed with the infection. The A/H7 infections that were reported here made up more than half of the cases in the vaccination and treatment program [131]. The findings highlighted the significance of effective surveillance, outbreak readiness, and pandemic preparation.

### 3.9. Clinical Findings in H7N3 Infection

In 2004, poultry in British Columbia, Canada, experienced an epidemic of the highly virulent AI H7N3 virus. Even while the improved surveillance found 57 people who fit the criterion of a suspected case, only two were found to have an AI infection. Conjunctivitis and a mild influenza-like sickness were among the symptoms experienced by the two patients. There were no HA inhibition or serum-neutralizing antibody responses in either of the two verified cases. A highly localized infection without the production of systemic antibodies was projected to be one cause for such an observation. However, in suspected instances, respiratory symptoms were more common than conjunctival symptoms. The genomic sequences of the two avian viruses from the source farms of the human isolates were consistent with the HPAIV, while one of the human isolates’ genomic sequences was compatible with the LPAIV. The existence of an insertion sequence in the human LPAIV isolate suggested that the HPAIV in poultry underwent a mutation to become the LPAIV, both of which were undetected when circulating among the birds on the source farm in question. The possibility that the human who died underwent a mutation from HPAIV to LPAIV was a less plausible scenario [132]. 

### 3.10. Clinical Findings in H3N2 Infection

A sporadic person-to-person transmission of swine H3N2 viruses that caused a mild influenza-like disease was reported in children in 2011. The condition was found to be brought on by the triple reassortant influenza A (H3N2) virus that occurred from swine carrying the matrix (M) gene from the pandemic 2009 influenza A (H1N1) virus. These viruses were regarded as the reassortant between a pH1N1 virus and an influenza A H3N2 virus originating among North American swine. None of the H3N2 virus-infected children needed hospitalization, and they all fully recovered after a brief episode of febrile respiratory illness [133].

## 4. Diagnosis of Avian Influenza Virus

The diagnosis of AI involves the integration of traditional approaches with developing technologies that are quickly evolving. Selection of a diagnostic tool may be based on variety of factors, including its suitability for the motive, technical simplicity, speed, diagnostic sensitivity and expense. Tests for the precise detection of the AI virus fall into two categories: those that demonstrate the virus directly and those that demonstrate the virus exposure indirectly by detecting a particular antibody. Direct detection involves both traditional viral infection culture and the use of quicker, more efficient methods that may identify certain viral antigens or nucleic acids such as the PCR assay, or the isolation of the virus in a cell culture. A brief description of some of the diagnostic methodologies has been detailed below:

### 4.1. Serology: A Diagnosis of Influenza Infection can Be Made Retrospectively Using Serologic Assays

The effective ELISA-based methodologies that offer on-the-spot detection properties and the possibility for the distinct identification of AI subtypes, as well as advancements in traditional agar-gel immunodiffusion and hemagglutination inhibition (HAI) techniques, are all parts of the antibody-based identification of the AI virus subjection [134]. Immunochromatography (IC), an antigen-based assay, is another crucial and fast diagnostic tool for the clinical detection as well as monitoring of IAVs [135,136]. 

### 4.2. Virus Isolation Test

**Figure 7:**
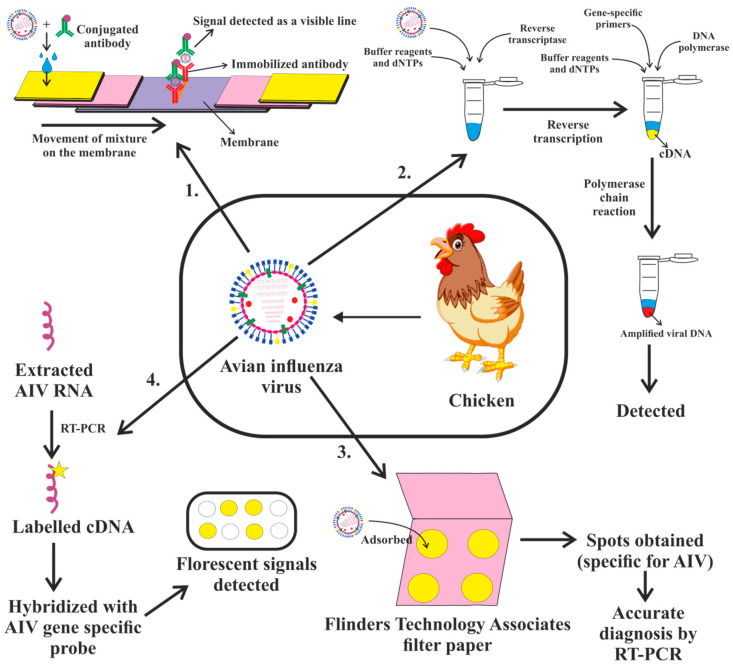
Different strategies for the detection of avian influenza virus. (1) Depicts immunochromatographic detection of AIV, (2) represents detection of AIV through RT-PCR technique, (3) depicts detection of AIV using Flinders Technology Associates filter paper technique and 4) depicts detection of AIV using DNA microarray technology (AIV = Avian influenza virus).

The concept is analogous to an ELISA sandwich. Nasal swabs from patients are mixed immediately with antibodies that are specific to viral antigens, colloidal gold, colored latex or a particular enzyme. The mixture is placed over a sample pad comprised of a nitrocellulose membrane and fixed viral antigen-specific antibody to collect the antigen. A line or dot appears as a positive signal when the antigen–antibody combination in the mixture moves on the membrane and makes contact with the immobilized antibody (Figure 7).

### 4.3. Rapid Influenza Diagnostic Tests

Microscopically, in the case of AI infections, edema and lymphocytic cell penetration may be observed, but secondary infections typically involve heterophilic to fibrinocellular infiltration. Enteritis and acinar cell decay are more normal in turkeys than in chickens. Lesions in susceptible birds caused by HPAIVs are more dramatic. There are edematous, bleeding, and necrotic lesions on the visceral organs and unfeathered skin. [137]. A record of many unforeseen deaths, frequently occurring within a few days, along with the classic lesions of cyanosis, edema and hemorrhage of the head and shanks, petechial hemorrhages of visceral organs, and hemorrhage and necrosis of mucosal surfaces, should trigger the beginning of confirmatory testing for the AIV [138]. As a quick and easy screening tool in addition to traditional H&E staining for the assessment of tissue lesions in environments with limited resources, indirect fluorescent antibody staining of tissues using a nucleoprotein-specific monoclonal antibody to identify the existence and localization of IAV has been demonstrated [139]. Species heterogeneity in the vulnerability and seriousness of lesions is particularly fascinating, as domestic ducks and migratory birds have been related to the preservation and spread of the Asiatic H5N1 virus [140,141,142]. 

### 4.4. Molecular Influenza Diagnostic Tests

In recent years, the number of methods for identifying nucleic acids, particularly the PCR-based diagnosis of AI viruses, has grown exponentially. Both standard RT-PCR and real time RT-PCR methods are widely utilized for influenza virus detection and subtyping based on genetic sequences in the HA gene and NA gene, as well as for observation and identification purposes [143]. The purified influenza A viral RNA is used to diagnose IAV. The viral RNA is reverse transcribed into cDNA by a reverse transcriptase, which is subsequently amplified by certain primers (Figure 7). In all eight of the IAV gene’s segments, the first 12 nucleotides of the 3’ terminus (Uni12) and the first 13 nucleotides of the 5’ terminus (Uni13) are conservative. The Uni12 and Uni13 sequences are multiplied many times by all IAV segments simultaneously. The Uni12 and Uni13 primers may be used in a RT-PCR test to detect IAV in human nasal swabs, and the subsequent sequencing analysis confers precise knowledge on viral subtypes [144,145,146,147,148,149]. 

The PCR test is time consuming and vulnerable to cross-contamination, since the post-PCR handling procedures require one to analyze the amplicon. Contrarily, the real-time PCR eliminates the requirement for post-PCR processing by allowing for the instantaneous identification of the PCR product during the amplification procedure. However, compared to the equipment needed for RT-PCR, this approach necessitates the need for specialized tools and materials, which are costly. Notomi et al. published a revolutionary nucleic acid amplification method known as loop-mediated isothermal amplification (LAMP) in 2000 that stimulates self-repeating strand-displacement DNA synthesis. LAMP is simpler than RT-PCR and may be finished in 30 to 60 min. More notably, LAMP may be performed in a water bath at a constant temperature range of 60 to 65 °C without the need for any expensive or specialized equipment [150]. It was established that the reverse transcription-LAMP technique amplifies only the RNA of the H5 subtype virus by utilizing viral RNAs taken from AIVs of the H1-H15 HA subtypes and human pathogenic respiratory viruses. The technique identified H5-HA genes in throat swabs taken from both people and wild birds [151,152,153].

A DNA microarray is an array of oligonucleotides, probes or DNA spots fixed on a surface. The technique is used for high-throughput, concurrent and broad genomic screening (Figure 7) [154,155]. Development of assays for identifying and subtyping influenza A viruses are just examples of how rapidly the microarray technology has advanced from its applications in basic research to detection and diagnostic formats. The technology, which makes use of immobilized capture oligonucleotides, enables the simultaneous detection of single nucleotide polymorphisms and thousands of other nucleotide sequences. Within 12 h, it has been demonstrated that a low density microarray with 15 capture oligonucleotides targeting the conserved influenza matrix gene can precisely identify the H1N1, H3N2, and H5N1 subtypes of IAVs [156,157,158,159,160]. 

The viability of the Flinders Technology Associates filter paper (FTA^®^ card) for AIV infection and the preservation of viral RNA for identification by the RT-qPCR, sequencing and DNA microarray test was examined by an investigation (Figure 7). It was found that AIV subtype H6N2 and HPAIV subtype H5N1 entirely lost their ability to infect within an hour of adhering to the FTA card at an ambient temperature. In addition, the viral RNA that was FTA-adsorbed remained sturdy for five months. Swab samples from experimentally diseased chickens were quickly detected on the FTA^®^ cards, enabling a precise and simple RT-qPCR identification of the H5N1 virus. While AIV RNA was found with less sensitivity than the direct evaluation of swab fluids, FTA^®^ cards were nevertheless appropriate for examining field samples. Additionally, the use of FTA^®^ cards can also enable a secure sample transfer for the molecular testing of AIV, thus obviating the requirement for continuous cold storage [161].

## 5. Immune Response to Avian Influenza Viruses

The main goal of an immunological response is to identify and get rid of the infection. Vertebrates have an immune system composed of two functional components, the innate and the adaptive, which vary in terms of how quickly they respond to pathogens and how they recognize them [162,163]. Pattern recognition receptors (PRRs) are germline-encoded receptors that identify pathogen-associated molecular patterns (PAMPs) present on infectious microorganisms, which have evolved with conserved molecular markers. These receptors are utilized in the initial responses of the innate immune system [162,164,165]. On the other hand, highly specialized antigen receptors, created randomly by gene rearrangement on T cells and B lymphocytes, are used by the more sophisticated adaptive immune responses [164,166]. The innate immunological response activates the adaptive immunological response and also affects the type of response.

The host’s immune response is imperative in understanding the pathogenesis of diseases caused by viruses and is the foundation for the creation of control measures [5]. The immune system’s response to illness with the AIVs and susceptibility to illness vary greatly among chicken species. The immunological mechanisms behind AIV resistance or susceptibility in avian species are unclear and likely rely on some variables, including but not limited to host genetics, isolate pathogenicity, infection dosage and bird immune state. Because the virus can harm the host so quickly and has evolved to modulate the host’s innate immune response, the virus is challenging for the immune system to combat. AIV also exhibits a high mutation frequency and the capacity to rearrange gene segments, which enable the virus to quickly change its antigenic makeup and escape the immune system’s adaptive response [167].

While a robust immune response is necessary for successful viral suppression, it must be finely controlled, since both in human and animal models an excessive and/or exaggerated inflammatory response has been linked to the worsening of tissue damage. The H5N1 virus such as H7N9 is of avian origin and generates a severe clinical presentation with a high death rate. The H5N1 virus is a good example of this double-edged effect. A “cytokine storm”, or the dysregulated rise of proinflammatory cytokines linked to H5N1 infections, is thought to be the primary factor contributing to the infection’s broad pulmonary tissue destruction [100,168]. The interleukin-6 (IL-6), interferon (IFN), tumor necrosis factor (TNF), and chemokines are examples of pro-inflammatory cytokines whose levels are markedly elevated during influenza virus infection [169]. The primary innate immune defense mechanism against viral infections depends on the degree of IFN production, and IFN is crucial in the early stages of the antiviral response [170,171]. When IFN production is dysregulated, it leads to inhibition of virus replication through the generation of antiviral mediators and exacerbation of the immunopathological destruction. Depending on the timing and intensity of the infection, there may be positive or negative outcomes due to these immune responses [172]. In addition, monocytes/macrophages and neutrophils are the primary innate immune cells engaged into the alveoli in the early phase of the infection following a viral infection [173]. In addition to being able to phagocytose the infected target cells, monocytes and macrophages can also differentiate into several subtypes that can release various cytokines. The most prevalent macrophage subtypes are classically activated macrophages (M1) and alternatively activated macrophages (M2) [174]. Depending on their quantities and maintenance times, pro-inflammatory cytokines secreted by polarized M1 cells early in an infection may have protective or immunopathological consequences [175]. M2 cells are involved in tissue healing and reducing inflammation. It has been observed that the ratio of M1 to M2 fluctuates continually until the pathogen is totally eradicated and the tissue healing approaches homoeostasis [176].

Four sources add to our current comprehension of the immunological response to AIVs, i.e., extrapolation from the knowledge of seasonal influenza viruses, information from in vitro investigations and animal models using AIVs, and analysis of the immunological reactions in patients infected with AIVs. By identifying PAMPs by PRRs, the innate immune system can identify viral illnesses. At least three different kinds of PRRs can detect influenza virus infection: TLRs, retinoic acid-induced gene I (RIG-I)-like receptors and nucleotide oligomerization domain (NOD)-like receptors [177]. 

### 5.1. Innate Immunity

An IV seeks to evade the innate immunological response to establish an illness as well as to propagate successfully in the body. The innate immunological response works for preventing and restricting viral replication inside the body. Additionally, it has a vital role in activating the adaptive immune response. The adaptive immune reaction is critical in clearing the infected cells along with the remaining influenza virus and resolving the infection, as well as in generating memory cells that have a crucial role in responding to the future influenza infection [178,179,180,181]. The innate immune system indulges in the early recognition of viruses, and is an important barrier in defending the infection caused by the viruses [182,183]. IAVs invade the mucus covering of respiratory epithelium and epithelial cells, and then disseminate to other immune and non-immune cells. In these cells, the virus is easily sensed by the recognition receptors; this results in the production of type 1 IFNs being triggered, which in turn is responsible for inducing the expression of numerous IFN-stimulated genes. These prevent the virus from replicating and spreading any further. Simultaneously, the generation of pro-inflammatory cytokines and chemokines also takes place. Pro-inflammatory cytokines, e.g., interleukin-1 β (IL-1β), IL-6, interleukin-18 (IL-18), and TNF, induce topical and systemic inflammation causing anorexia and fever. In addition, the production of cytokines by the airway epithelium in response to viral replication draws innate immune cells to the infection site. These leukocytes, including neutrophils, monocytes, macrophages, DCs, eosinophils, natural killer (NK) cells, and T lymphocytes, become activated in response to IAVs. This action inhibits the virus and safeguards the airway epithelium, while also inducing the adaptive immune response [182,184].

Ex vivo research on macrophages and the human monocyte-obtained primary airway cells has examined the innate immune responses to IAVs. Chemokine and cytokine upregulation has been observed in respiratory epithelial cells infected with H5N1 viruses. The induction of proinflammatory cytokines has also been observed as the result of H5N1 infection in primary endothelial cells as well as human monocyte-derived macrophages [5]. Furthermore, IAVs have also been reported to translate the nonstructural protein 1 (NS1), a multifunctional immunosuppressive factor, which leads to the inhibition of antiviral innate immune responses, notably the interferon (IFN)—Janus kinase (JAK)-signaling transducer and activator of the transcription (STAT) signaling pathway [185]. In both in vitro and in vivo investigations, studies have discovered that SSU72, a gene loop regulator at TTSs, regulates the expression of genes related in the innate immunological response and plays a critical role in transcriptional readthrough (TRT) regulation. To sustain the development of gene loops and transcriptional directionality, SSU72 binds to the promoter and terminator regions. Recently, an investigation found that the H5N1 nonstructural protein 1 (NS1) may bind to SSU72 directly and inhibit its expression. On the other hand, the SSU72 overexpression reduced lung damage and protected animals infected with H5N1 from inhibiting the antiviral gene expression. Hence, it was emphasized that SSU72 might act as a possible therapeutic target in the treatment of AIV infections [186].

#### 5.1.1. Interferons

The antiviral response is triggered by double-stranded RNA (dsRNA) by-products of IAV multiplication and takes place swiftly, within minutes. RIG-I, the melanoma differentiation-associated protein 5 (MDA-5), and other proteins that have N-terminal caspase recruitment domains (CARDs) and C-terminal DExD box RNA helicase domains, recognize dsRNA [187]. When dsRNA binds to the helicase domain, it initiates a second contact with the CARD-containing protein IPS-1, which in turn activates the IkappaB kinase (IKK)-related kinases TBK-1 and IKKe, whichphosphorylate interferon regulatory factor 3 (IRF3) when it comes to the IKK pathway [188]. IFR3 belongs to one of the nine members of the IRF transcription factor family and is crucial for IFN expression [189]. The interaction of the transcription factors, the nuclear factor kappa- (NFKB), ATF2/c-Jun and IRF3 on the positive regulatory domain enhancer element of the IFNB promoter and the interferon-stimulated response element (ISRE) on the promoters of a subset of IFN-stimulated genes (ISG), results in the induction of the IFN transcription [190,191,192]. The ISG factor-3 (ISGF3) is produced by the JAK-STAT pathway being activated by the binding of secreted IFNs to type I IFN receptors on cell surfaces. STAT1, STAT2 and IRF9 are the components of the heterotrimeric complex known as ISGF3 [193]. A positive feedback mechanism causes IFN- and multiple ISGs to be transcribed when the ISGF3 translocates to the nucleus, thus amplifying the response. Viral proteins commonly aim to suppress these stages of IFN induction, amplification and effector action. Previous studies have demonstrated that AIVs are susceptible to type I IFN’s antiviral actions [194,195,196,197]. It has also been reported by research studies that only RIG-I is required to produce IFN in response to IAV infection using cells and animals defective in either RIG-I or MDA-5 [198,199,200]. Earlier research reports have also demonstrated that pre-exposing chicken cells to IFN significantly lowered the incidence of AI infection [201]. IFN’s effect is therefore highly dependent on the time and degree of its expression. 

#### 5.1.2. Antigen-Presenting Cells

DCs and respiratory macrophages are the primary immune cells to recognize and respond to IAVs, which are crucial for maintaining a balance between innate and adaptive immune responses [202,203,204]. According to several findings, IAVs replicatesuccessfully in human and mouse macrophages, as well as in DCs. However, other investigations have labeled these infections as being “abortive” or “dead end” [205]. The course of infection is thought to be influenced by variations in the viral strain and subsets of macrophages and DC (produced from the lung and from the blood of bone marrow). The majority of seasonal IAV and LPAIV infections are abortive, which aids in efficient host protection. Some HPAIV strains may productively infect macrophages and DCs, which plausibly impact viral spread, amplification and ultimately pathogenicity and immunogenicity [96,206]. 

#### 5.1.3. Natural Killer Cells

The numbers of NK cells have been reduced in the blood of human patients with a severe influenza infection [207,208]. NK cells have been demonstrated to be necessary for the clearance of IAV in in vivo investigations using mice. Intriguingly, the increased NK cell activation in chicken lungs following the H9N2 virus infection has also been documented. Contrarily, the infection with H5N1 HPAIVs has been found to lead to a reduction in the stimulation of lung NK cells, suggesting that this may be one of the processes underlying the infectivity of H5N1 HPAIVs [209].

#### 5.1.4. Inducible Antimicrobial Components

The enthusiastic reactions brought on by the IAV invasion include the involvement of several different proteins. Acute-phase proteins (APPs), the C-reactive protein (CRP), serum amyloid A, collagenous lectins, alpha-macroglobulin families and antimicrobial peptides are just a few of the families of proteins that make up these innate inhibitors [210,211]. Collectins contain carbohydrate recognition domains (CRDs) that bind to the mannose-rich glycans on the viral HA and sporadically to the NA to regulate a variety of anti-IAV actions. These include decreasing the IAV hemagglutination and NA enzyme activity, reducing viral virulence, preventing virus aggregation and increasing neutrophil IAV uptake [212,213]. 

#### 5.1.5. Polymorphonuclear Leukocytes

Granulocytes and polymorphonuclear leukocytes are crucial host protection cells during the innate immunity phase. By phagocytosing microorganisms, stimulated granulocytes destroy the eaten organisms by producing a combination of bactericidal peptides, defensins, proteolytic enzymes, harmful oxygen radicals and myeloperoxidase [214,215]. Viruses can activate granulocytes by direct binding or through binding with antiviral antibodies which results in antibody-dependent cytotoxicity. Simpler influenza inflammatory infiltrates with a predominance of mononuclear leukocytes and a lesser number of polymorphonuclear leukocytes are caused by viral multiplication in the lung epithelium. However, the lung inflammation was linked to pathological alterations in alveolar macrophages and neutrophil migration following infection with extremely virulent IAV strains, such as the lethal H1N1 1918 HA/NA:Tx/91 in a mouse model [216]. 

#### 5.1.6. Role of Cytokine Storm in Innate Immunity Response

The intensity and prognosis of an influenza infection may be significantly influenced by the dysregulation of the innate immune response [96,173]. According to several reports, tissue damage and excessive mortality in mice, macaques, ferrets and in vitro infection models are caused by the dysregulation of lungs’ cytokines and chemokines [217,218,219,220]. Higher levels of many cytokines and chemokines have been linked to more a severe AIV illness and poorer survival outcomes [221,222,223]. The synergistic interaction of many pathways, including the RIG-I, TNF and type I IFN pathways, causes an increase in the levels of IFN and pro-inflammatory cytokines [224]. TNF-α is a crucial cytokine in cytokine storms and is probably accountable for the worsening of disease seriousness [225]. The AIV infection causes the release of several ISGs, IFNs, pro-inflammatory cytokines and chemokines; nonetheless, the virus manages to avoid the host’s immune system and replicates effectively in the host tissues. This avoidance of the host’s innate immunological system is carried out through interfering with the IFN induction, IFN-inducible signaling and IFN-mediated effector actions [202]. The suppressor of cytokine signaling (SOCS), a powerful endogenous inhibitor of TLR signaling and IFN signaling, is one such mechanism. The up-regulation of SOCS1 and SOCS3 genes reduces the host’s ability to fight the virus and creates a pathway for effective virus replication in lung tissues. The enhanced pro-inflammatory cytokine expression is brought on by the elevated viral load in the lung cells, and in chickens, this hyperinflammatory response results in severe multiple organ failure and rapid death [226,227,228]. Monocytes/macrophages are the primary cells drawn into the alveolar space in the first phase of the viral infection. They produce more cytokines and chemoattract more immune cells to the lesion site. However, they are also prone to influenza virus infection. Monocytes/macrophages play a significant part in viral clearance, as their depletion influences the immunopathology [173].

#### 5.1.7. Role of Toll-Like Receptors (TLRs) in Innate Immunity Response

Toll-like receptors are PRRs that recognize pathogens’ molecular patterns, including lipids, proteins and nucleic acids [229]. In order to trigger innate immune responses in mammalian hosts, TLRs are essential for the identification of microbial pathogens [230]. Inflammatory cytokines and reactive oxygen and nitrogen intermediates are produced when pathogens recognize TLRs. Several TLR genes have been found in chickens: TLR1A, 1B, 2A, 2B, 3, 4, 5, 7, 15 and 21. By activating various TLRs, the H9N2 infection of chickens may cause the trachea, lung and intestine cells to produce inflammatory cytokines [230]. Similarly, ten TLR family members (TLR1-TLR10) have been found in humans, and several of them seem to be capable of identifying particular microbial compounds such as the lipopolysaccharide (LPS), bacterial lipoproteins, peptidoglycan and bacterial DNA [230]. The transcription factors, the IFN regulatory factor 7 (IRF7)/IRF3 or nuclear factor-B (NF-B), are activated as a result of the activation of TLR signaling by TLR3 and TLR7, which detect the avian influenza viral RNA and trigger the development of type I IFNs and pro-inflammatory cytokines [231]. TLR10 has been demonstrated by Lee et al. to be involved in innate immune responses following influenza virus infection. Given the significance of innate immunological sensing receptors in host defense and pathogenesis, the functional relevance of TLR10 in influenza infection raises the likelihood that this receptor is involved in a variety of other viral and possibly microbial diseases [232]. In geese infected with the H5N1 HPAIV, Wei et al. noted the activation of the TLR7 MyD88-dependent pathway, which generates interferon-stimulated genes [233]. An infection with the avian influenza also causes the activation of other TLRs (TLR 1, 2, 4, 5 and 15), among which TLR15 is regarded as being avian-specific [234,235]. AIV-infected chickens have recently been treated with TLR ligands (polyI:C and CpG) as adjuvants or antiviral agents; the results revealed the induction of the innate immune gene expression [236], antibody and cell-mediated immune responses [237], and significantly decreased AIV shedding [238].

It is interesting to note that the genomes of birds appear to lack several immunological genes essential for innate responses. The absence of functioning TLR8 in the genomes of chickens hampers their ability to recognize intracellular bacteria and RNA viruses, particularly IAV, and may be related to the elevated death rates observed during HPAIV infection. Similarly, ducks lack TLR8 and are resistant to HPAIV infections. Importantly, no single innate component, cell or pathway contributes to the infection’s cause, since so many different reactions and components go into fighting influenza. Hence, future studies on innate responses will not only advance our fundamental knowledge of disease resistance but also facilitate intervention approaches to increase the early protection against viral entry and spread, as well as the initiation and intensity of adaptive immunological responses [167].

### 5.2. Adaptive Immunity

**Figure 8:**
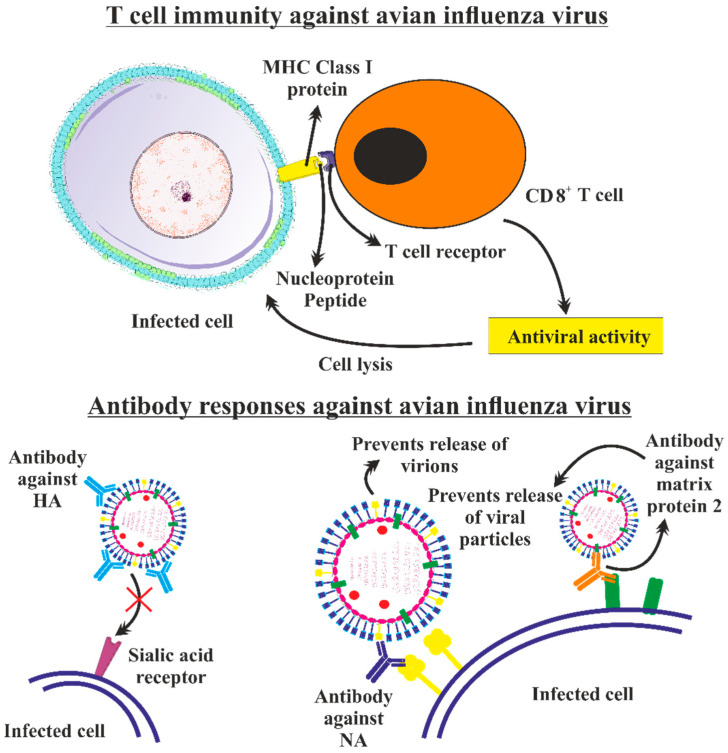
When CD8^+^ T cells with specificity for viral proteins such as nucleoprotein (NP) or the RNA polymerase proteins such as polymerase basic protein 2 (PB2) and polymerase acidic protein (PA) recognize viral peptides presented by MHC class I molecules, which leads to the release of cytokines with antiviral activity that mediate cytolysis of the infected cell; HA-specific antibodies stop the fusion of cells or impede viral attachment, thus preventing cell infection; NA-specific antibodies attach the virus to the cell and circumvent the release of virions; M2-specific antibodies attach virus to the cell and stop viral particles from escaping into the extracellular fluid.

**Figure 9:**
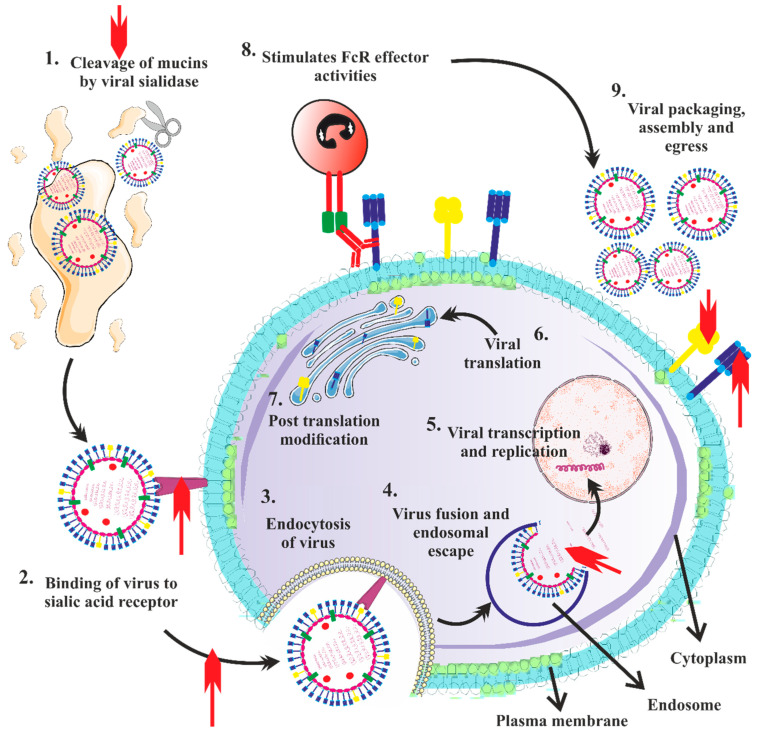
Schematic diagram of life cycle of influenza virus and possible targets (shown by red arrows) for defensive antibodies. (1) The viral neuraminidase (NA), an enzyme that cleaves mucins to gain access to respiratory cells, facilitates viral entrance in the respiratory tract. Antibodies that disrupt the enzymatic activity of NA, such as anti-NA antibodies or anti-hemagglutinin (HA) antibodies, may be able to stop this process. (2) The head of HA binds to sialic acid receptors on the surface of cells, thus mediating viral entrance. Endosomal escape is then accomplished by the fusing of the viral and endosomal membranes. In addition to blocking this interaction, antibodies that bind to the HA head domain can provide sterile protection from infection. (3) Alternately, blocking HA-neutralizing antibodies can prevent the internalization of the influenza virus after post-binding; or (4) its capacity to fuse and escape from the endosome. (5) For viral transcription and replication, ribonucleoproteins from the virus (vRNPs) are transported into the nucleus; (6) mRNAs are exported to the cytoplasm to be translated into viral proteins; (7) for post-translational modification and subsequent presentation on the cell surface, HA and NA are transported to the Golgi; (8) anti-HA stalk antibodies identify HA on infected cells’ surfaces and activate Fc-mediated effector mechanisms such as antibody-dependent cellular cytotoxicity, which can then be used to target and destroy the infected cell. (9) It is at the plasma membrane that viruses are packaged, assembled, and expelled. Antibodies that inhibit egress, such as anti-HA or anti-NA antibodies, may also target this pathway. Anti-NA antibodies can do this by inhibiting the release of fresh virions from the surface of infected cells or by forcing fresh virions to cluster in the absence of NA activity. The figure is adapted from Krammer, 2019 [262].

In the case of AIV infection, the attachment of the virus within the host happens mostly because of interactions between the HA protein and host sialic acid residues located on cells covering the mucosal surface As a result of this, the virus then internalizes and infects the host. A decrease in the pH caused by the virus’s endosomal containment and maturation is required for the virus to uncoat. Through interactions between the single-stranded viral RNA genome TLR7 and endosomally positioned TLRs, the host’s innate immunological reaction is triggered at this stage. A cascade of cytokines and interferons are produced when the RNA agonist activates TLR7 to prevent or reduce viral multiplication and draw effector cells to induce adaptive immunity [239,240]. Major histocompatibility complex (MHC) class I molecules modify and express viral proteins as the virus replicates in the cytoplasm. CD8^+^ lymphocytes then transmit viral proteins to cytotoxic T-lymphocytes (CTLs), which can destroy virus-infected cells. (Figure 8) (Figure 9) Simultaneously, professional APCs including macrophages, DCs, and B cells pick up newly released virions. The processed viral peptides are recognized by CD4^+^ T-helper cells through the production of MHC class II molecules. When B cells receive viral antigen from these CD4^+^ lymphocytes, they develop antibodies against the viral antigens. The generation of strong, targeted B and T cell immunity in the host, developing with a memory response, as has previously been demonstrated with H7N9 and pH1N1 infections [241,242,243,244,245,246], as well as humoral immunity [244] for H5N1 infections, is necessary for the endurance and recovery from AIV infection.

#### 5.2.1. T Cell Immunity against Avian Influenza Virus

For many years, much attention has been given to CD8^+^ T cell immunity, and extensive research has been done to comprehend the mechanism of action of CD8^+^ T cell responses against different pathogens in animal models. Additionally, their importance in heterosubtypic protection has also been studied in the past 40 years [247]. Animals exposed to an IV acquire a protective immunity against the exposure to a virus of a different subtype that is accompanied by a decrease in viral replication [248,249]. Usually, the protection was exhibited in the truancy of cross-reactive antibodies and linked with cross-reactive CTLs. These CTLs were found in the circulation, spleen, and/or draining lymph nodes. Human CTLs produced against seasonal influenza A viruses have also been shown in in vitro tests to be able to detect and respond to HPAIVs and the novel A/H1N1 virus [250,251]. The unequal age organization of the pandemic H1N1 and HPAI H5N1 cases further demonstrates that the already existing immunity brought on by seasonal IAV infections, which may be lacking or not functioning as well in youngsters, may help to protect against a heterologous infection [252,253]. Consequently, to mediate the immunization against new avian IAVs, pre-existing stores of cross-reactive memory T cells are specifically crucial. It was evident from a longitudinal analysis of peripheral blood mononuclear cells (PBMCs) taken from H7N9-infected people that recovery from severe illness was mediated by several immunological effectors, predominated by CD8^+^ T cells [241,254]. In example, people who recuperated from the avian H7N9 IAV illness had robust IFN-^+^CD8^+^ T cell responses, in contrast to those who died due to illness and had some or no IFN-producing cells, extending the activation of exhausted PD-1-expressing CD38^+^HLA-DR^+^ CD8^+^ T cells. The transcriptomes of the stimulated CD38^+^HLA-DR^+^ CD8^+^ T cells demonstrated substantial variations between fatal and nonfatal patients along with this. Different gene expression patterns associated with a clinical outcome were found using a single-cell RNA sequencing analysis, with a differential segregation specifically at the initial time periods following the illness start. During the lethal H7N9 epidemic, the heat shock protein DNAJB1, IFN-induced transmembrane protein 3 (IFITM3), lactate dehydrogenase A (LDHA), and programmed cell death protein 5 (PDCD5) genes demonstrated the most obvious differences. Additionally, the recovery was linked to a vigorous development of cross-reactive CD8^+^ TCR-αβ clonotypes, while delayed and restricted expansions were observed in fatal infections [254]. Moreover, the evidence has demonstrated that patients who are released from the hospital within two to three weeks show early and strong CD8^+^ T cell responses that are specific for H7N9, whereas patients who require lengthy hospital stays show delayed engagement of CD8^+^/CD4^+^ T cells and antibodies at the same time, which is further impeded by strong NK cell responses. In contrast, individuals who died showed little or no T cell activation and a limited immunity specific to influenza [241]. Table 2 explains different research studies evaluating the human T cell protection towards AIVs.

The importance of the duck cytotoxic T cell response in eradicating the H5N1 infection in vivo was suggested by an investigation through the detection of significantly more CD8^+^ cells and the increased expression of cytotoxicity-related genes, such as granzyme A and IFN, in PBMCs from 5 to 9 days post-infection in mallard duck infection experiments. Interestingly, the CD8^+^ cell population of PBMCs was markedly increased in infected ducks from 7 to 9 days post-infection compared to uninfected ducks. As a result, both in vivo and in vitro, the high duck CD8^+^ cells provided a potentially efficient immune response to the H5N1 AIV infection and offered novel perspectives and insights for creating efficient H5N1 AIV vaccines [255]. Furthermore, Hao et al. developed a multi-parameter flow cytometry to study the innate and adaptive cellular immunological responses in chickens following intranasal infection with low pathogenic H7N9 AIV. After H7N9 infection, it was discovered that NK cells and KUL01^+^ cells considerably increased, particularly in the lung. The KUL01^+^ cells also raised MHC-II and CD11c expression. Additionally, CD8^+^ T cells and γδ-T cells considerably increased in percentage and quantity and demonstrated an activated phenotype, and a considerable overexpression of the CD25 expression was also observed in the lung, but not in the spleen or blood. Furthermore, the percentages or/and quantities of B cells increased in the lung but dropped in the blood and spleen, indicating that these cells may have been drawn from the periphery following the H7N9 infection [256]. Overall, these results offered compelling evidence that influenza-specific CD8^+^ T lymphocytes play a critical part in modulating human recuperation from the avian IAV infection.

Recently, a study also examined the impact of exacerbated innate immunological responses on the impairment of subsequent T cell adaptive immune responses in the lungs. With the use of recombinant H1N1 and H5N1 strains that share six internal genes, it was demonstrated that the H5N1 infection in mice resulted in stronger activation and enhanced movement of lung DCs to the lung-draining LNs, leading to greater amounts of virus-specific T lymphocytes in the lungs. In addition, the H5N1-infected mice had slower and less effective viral clearance than the H1N1-infected animals, despite strong T cell responses in the lungs. Furthermore, higher amounts of inhibitory signals, such as enhanced PD-1 and interleukin-10 (IL-10) production by cytotoxic T cells, were discovered in H5N1-infected mice. These inhibitory signals may have slowed the viral clearance of the H5N1 infection by inhibiting the T cell activity in vivo. In particular, animals infected with H5N1 had less tissue-resident memory T cells than mice infected with H1N1, yet H5N1 was still resistant to a challenge from the H3N2 virus, despite having fewer tissue-residing memory T cells [257].

However, since each person possesses some immunity, the interpretation of these studies’ findings is difficult. Hence, it is not feasible to comprehend the exact function of T cells in lessening the disease’s severity [258,259,260,261]. Additionally, all human studies must consider the various subsets of CD8^+^ T cells that are present in peripheral blood. The majority of the induced CD8^+^ T cells are deposited in peripheral tissues, although their ability to recirculate is influenced by T cell differentiation, signals that encourage recirculation and homing characteristics. A different proportion of circulating and tissue resident CD8^+^ T cells can be induced by vaccination and infection. As in the case of the respiratory influenza infection, the analysis of blood for a CD8^+^ T cell response is not precise, as the majority of effector CD8^+^ T cells become accumulated in peripheral tissues [247]. The most of the changes that occur in CD8^+^ T cells in humans following vaccination or in a natural influenza infection are still not known and should be clarified for adequately understanding their role in controlling influenza.

**Table 2:** Research studies evaluating human T cell immunity towards avian influenza viruses.

S. No.	Subtype of Avian Influenza Virus	T Cells	Important Remarks	Reference
1	H5N1	CD8^+^ T cells	The importance of the duck cytotoxic T cell response in eradicating H5N1 infection in vivo was suggested through the detection of significantly more CD8^+^ cells and increased stimulation of cytotoxicity-associated genes, such as granzyme A and IFN, in PBMCs from 5 to 9 days post-infection in mallard duck infection experiments.	[255]
2	H5N1	CD4^+^ T cells, CD8^+^ T cells	The impact of exacerbated innate immune responses on the impairment of subsequent T cell adaptive immune responses in the lungs was reported.	[257]
3	H7N9	CD8^+^ T cells, γδ-T cells	Considerable increase in CD8^+^ T cells and γδ-T cells’ percentage and quantity and a considerable overexpression of CD25 expression was observed in the lungs.	[256]
4	H7N9	CD38^+^HLA-DR^+^ CD8^+^ T cells	Those who recuperated from avian H7N9 IAV infection were reported to exhibit strong IFN-γ^+^CD8^+^ T cell responses, whereas those who died from infection had some or no IFN-γ-producing cells and showed protracted activation of exhausted PD-1-expressing CD38^+^HLA-DR^+^ CD8^+^ T cells.	[254]
5	H5N6	CD8^+^ T cells, CD45RA^+^ CCR7^-^ T cells	A thorough examination of the IFN-γ^+^ T cell response in the survivor revealed a preference for CD8^+^ T-cell-mediated immunity and an increase in virus-specific effector T cells (CD45RA^+^ CCR7^-^) between 10–18 days following the beginning of symptoms.	[31]
6	H7N9	CD8^+^ T cells, CD4^+^ T cells	High numbers of CD8^+^ T cells and CD4^+^ T cells were corresponded with better therapeutic consequences in H7N9 patients.	[235]
7	H7N9	CD8^+^ T cells	Early, strong CD8^+^ T cell responses that were specific for H7N9 were reported for patients who were released from the hospital within two to three weeks, whereas delayed enagagement of CD8^+^/CD4^+^ T cells and antibodies at the same time, which was further delayed by strong NK cell responses, were reported in patients who required lengthy hospital stays.	[241]
8	H7N9	CTLs’ response to PBMCs obtained from healthy indigenous people from Australia	Mutations that prevent CTL recognition as well as conserved immunogenic peptides that can trigger potent CTL responses against any human IAV, including the H7N9 virus, were reported.	[236]
9	H7N9	CD8^+^ T cells	It was determined that CD8^+^ T cells against seasonal influenza viruses have significant cross-reactivity with the new H7N9 virus, in addition to recognizing specific H7N9 variant epitopes.	[242]
10	H3N2, H1N1	CD4^+^ T cells	A correlation between lesser viral shedding and milder disease and preexisting CD4+, but not CD8+, T lymphocytes reacting to influenza internal proteins was reported.	[259]

#### 5.2.2. Antibody Responses against Avian Influenza Virus

The developing pandemic viruses quickly spread throughout the human population due to a lack of population immunity. However, they evolve into seasonal viruses within a few years, typically resulting in epidemics with lesser death rates than pandemics. Ubiquitous illness during the pandemic led to extensive population immunity, mostly in antibody reactions against HA and NA (Figure 8) [262]. It has long been recognized that antibody responses to the IV surface glycoproteins, notably HA, are protective against IV infection. Specific antibodies against HA have been established as a means of defense. Serum antibodies were found to be protective, even in the 1933 report that was the first to describe the isolation of the IV. The primary factor that permits developing pandemic viruses to rapidly disseminate all over the entire populace is the absence of population immunity based on antibodies [263]. The developmental strain applied by immunizer reactions (along with different elements and irregular occasions), generally from regular disease, drives the virus to modify its surface antigens, normally by initiating the point mutation, in a cycle called the antigenic drift. Significantly, the IV HA, particularly the globular head region, exhibits significant plasticity and is extremely adaptable to these modifications. The primary reason that IV vaccines must be updated annually is the antigenic drift. The effectiveness of the vaccine decreases dramatically if the vaccine’s virus strains are not antigenically compatible with the virus strains in circulation. This obviously also applies to novel, antigen-shifted pandemic viruses, for which vaccines that are antigenically suited are required [264]. A schematic diagram of the life cycle of the influenza virus and possible targets for defensive antibodies is pictorially depicted in Figure 9.

Therefore, the significance of the antibody response to IV cannot be overstated. Notably, innate immune responses and T cell responses are crucial for inducing strong antibody responses and greatly contribute to the protection against influenza viruses. Long-term research has been conducted on influenza virus-specific antibody responses. Particularly, in recent years, with the advancements in the development of technology that make it possible to analyze the antibodies created by individual human B cells, we have made enormous strides in our knowledge of the antibody response to IV [265]. Although the exact processes are unknown, it is known that antibody responses elicited by natural illness are often more extensive and durable than antibody reactions induced by vaccination. To enhance existing vaccinations, it would be helpful to understand these pathways. We have also recently found human antibodies that specifically target IV and are widely neutralizing. The key to achieving the purpose of a universal influenza virus vaccination may lie in understanding how these antibodies are produced. The importance of the antibody response to the IV cannot be overstated as a result. Notably, innate immune reactions and T cell reactions are essential for the generation of powerful antibody reactions and play a critical role in guarding against IVs [266,267,268,269,270,271,272].

## 6. Vaccine Development

It is evident that a continuous threat to world health is posed by illnesses that can spread from animal pools. IAVs have a variety of characteristics that make them a universal hazard. Because of the human population’s lack of immunological knowledge about these viruses, their subdivided genome and mistake-susceptible replication mechanism might result in the generation of new reassortant viruses [273,274,275,276,277]. Additionally, the fact that IAVs may spread to people, domestic animals and aquatic birds raises the possibility of reassortment and the eventual creation of new viruses. Vaccination is an effective methodology to circumvent the development of AIV infections. Seasonal flu vaccinations are safe and lessen the severity of yearly flu outbreaks. New influenza vaccinations are being developed to be prepared for potential pandemic IAV outbreaks in the future. It is possible to identify novel viral targets for vaccine development using the knowledge of the immune responses particular to influenza [278]. It may be useful to gain insights into the immune responses particular to influenza to identify new viral targets for vaccine development. Most of the vaccines used to date target the viral surface hemagglutinin (HA). The vaccines need to be updated, as the surface HA mutate easily via reassortments or antigenic drifts. Alternative approaches have been investigated due to the drawbacks of the traditional method of cultivating the virus in specific-pathogen-free embryonated hen eggs and a relatively new method of the cell culture of a virus to produce a vaccine. It has been demonstrated that recombinant HA-based vaccines can induce neutralizing antibodies against AIV. However, antibodies induced by a specific strain or subtype of the influenza virus are mostly ineffective in neutralizing other strains or subtypes. Due to the virus’s constant mutations, vaccines also needed to be updated after a certain period. Therefore, researchers are directing their efforts towards the development of universal influenza vaccines (UIVs) possessing a broad spectrum for neutralizing various influenza subtypes or strains [55]. 

### 6.1. The Objective of a Pandemic Influenza Vaccine

The enormous threat that HPAIVs and LPAIVs offer to human health is highlighted by the recent rise in reports of direct AIV transmission to people as well as the continuing H5N1 IV epidemic in avian species and humans in various countries. Since it is hard to project which subtype of AIV will give rise to the next human pandemic, the ideal vaccine would generate an immunological response that saves the host from illness with a wide variety of IVs from the same or other subtypes [279,280]. To circumvent the immune response, IVs’ HA and NA glycoproteins undergo genetic and antigenic mutation [281,282]. While cell-mediated immunity plays a significant part in the removal of human IVs, systemic or mucosal sites of illness that have neutralizing antibodies particular for the HA glycoprotein offer rapid protection against IV infection [283]. Although antibodies targeting the NA glycoprotein do not eliminate virulence, they do impede the generation of other virus molecules, which is a process that necessitates viral NA proteins, and limiting viral reproduction. As a result, NA-specific antibodies can lessen the severity of the illness. Human IVs have epitopes on their NP, PB2 and PA proteins that CTLs can recognize. Considering this, if a virus with a novel HA and/or NA glycoproteins infects the human population, the cell-mediated immunity conducted against the more conserved interior proteins may offer protection in the case of a pandemic [284,285]. The principle behind the presently approved vaccinations against human IVs is to induce protective and particular antibodies for the HA glycoprotein of the strain that is expected to cause an epidemic. 

AIV causes the induction of heterosubtypic immunity, which is predominantly correlated with the cross-reactive T cell induction [286]. Most of the cross-reactive T cells, mainly CD8^+^ CTLs, are focused on conserving the internal proteins, e.g., matrix proteins, polymerase complex proteins or nucleoproteins [287]. Seasonal AIV influenza infection induces CD8^+^ T cells, which has also been reported to cross react with viruses of A/H5N1, A/H7N9 and A/H1N1subtypes [288]. This cross-reactive T cell response induction seems to be an appealing action of UIVs [10]. Some vaccine delivery systems suitable for achieving this purpose are the live-attenuated vaccine, DNA vaccines, vector-based vaccines or virus-like particles (VLPs). VLPs consisting of NA, HA and M1 have been demonstrated to induce significant cell-mediated immunological responses than whole-inactivated vaccines in mice [289,290]. During phase 1 clinical trials, H5-specific T cells were elicited by the DNA vaccine encoding H5, HA and M2 in most subjects [10].

### 6.2. Types of Vaccines

Lately, live-attenuated influenza vaccines (LAIVs) and inactivated virus vaccines (IIVs) for the pandemic influenza are being increasingly developed. These vaccines are based on technology that has been granted a license for use in the current seasonal human influenza vaccinations. Live viral vectors producing IV proteins, DNA vaccines and other platforms are also being formulated for vaccinations, and the preclinical research has demonstrated potential for these approaches [291].

Different types of vaccines being developed and utilized against various subtypes of AIVs are discussed below.

#### 6.2.1. Inactivated Virus Vaccines 

IIV formulations can include split-virion or sub-unit immunizations as well as whole inactivated viral vaccines (WIV), each with benefits and drawbacks. WIV vaccines are frequently chemically inactivated and highly immunogenic because of their crude generation method and consequent activation of innate immunological signaling paths by residual virus RNA [292]. Relying on the inactivation method utilized, WIV vaccines can maintain the structural robustness of the HA and NA, the two key targets for neutralizing antibodies [181]. WIV vaccines contain internal antigens, which may increase cross-reactive T cell responses to conserved viral proteins such as nucleoprotein. However, WIV vaccines have not been preferred in recent years due to their higher reactogenicity as compared to formulations that are more extensively purified. Due to production processes that enrich for HA content, immune reactions to the hindmost vaccines are almost solely directed against HA [293,294,295,296,297]. However, it has been observed that a later IIV boost induces a strong antibody response even while the first vaccination only produces a mild or undetectable antibody response. The various prime-boost approaches should be compared side by side, and it should be investigated whether DNA and adenovirus-vectored vaccines also cause strongly localized germinal center reactions that are remembered systemically following the IIV boost [298,299].

#### 6.2.2. Live-Attenuated Influenza Viruses

The fundamental idea behind LAIVs is that the highly attenuated vaccination viruses are supplied intranasally and are only capable of reproducing in the URT due to mutations in the interior protein genes of the vaccine donor virus, which are temperature-sensitive and attenuating [300,301]. The LAIVs can only undergo some limited multiplications in the colder URT, and the viral spreading to the lungs is circumvented by cold-adaptation [302]. Such cold-adapted viruses are licensed and utilized in the USA and are created via the reassortment of a cold-adapted viruses with seasonal IAVs. Cold-adapted vaccines have been developed and evaluated in clinical trials against A/H5 viruses, which can stimulate a strong CD8^+^ T cell response in mice and ferrets [303]. These vaccines can elicit appreciable CD8^+^ T cells responses and protect animals from homologous as well as heterologous viruses [304]. During the Phase 1 trials, it was revealed that, though LAIVs are unable to produce a robust antibody response, a long-term immune memory is primed by them. This can be boosted by administering an inactivated vaccine 1–5 years later [303]. These vaccines successfully induce T cell responses focused on internal proteins. These HA-particular T cells were reported to induce the restricted cross-reactivity with HA of A/H5N1 [305]. Each LAIV is a reassortant virus made up of six interior protein genes from the vaccine donor virus, plus HA and NA gene segments taken from a wild-type IV. One potential benefit of creating LAIV for pandemic IVs is that the vaccine induces systemic and mucosal humoral and cellular immunological responses. Although limited, the LAIV replication is enough to cause systemic and mucosal antibody and T cell responses that guard the host against further illness [306,307]. The goal of the formulations of both IIVs and LAIVs is to induce protective antibodies directed toward HA and, to a slighter extent, NA. There are safety worries with the utilization of LAIV vaccines with avian HAs, even though they have a higher potential to produce cross-reactive immunity than IIVs, because it could be contended that immunization might enable reassortment if the recipient became concurrently infected with a seasonal IAV that is circulating in the population [308,309,310]. Additionally, not all population groups can benefit from LAIV vaccinations.

#### 6.2.3. Vector-Based Vaccines

Vector-based vaccines (recombinant poxvirus and adenovirus) are reported to be a promising vaccine candidate [311]. The majority of viral vectors are also known as “live” vaccines. Excellent safety profiles of these vaccines can be owed to their complete replication-deficiency in humans, even in immunocompromised individuals. Modified vaccinia virus Ankara (MVA) is a well described vaccine vector candidate [312]. This was originally manufactured as a replication-deficient smallpox vaccine [313]. MVA produced the endogenous antigen in infected cells, which resulted in the efficient modification and presentation of antigens, thus stimulating the antigen-particular B cell and T cell reactions. In addition, the development of the recombinant-modified vaccinia virus Ankara (rMVA) by inserting genes, which encoded the desired antigen into the viral genome, is an uncomplicated process. As a result, numerous rMVA vaccine contenders which express IAV M1, M2, NP, PB2, HA and NA have been effectively examined in ferrets and mice, as well as in macaque vaccination challenge experiments [314]. The rMVA vaccines, on which HA genes from various A/H5N1 viruses have been expressed, have been developed and studied for their defense efficacy against viruses which belong to different antigenic clades in animals [315]. 

Furthermore, adenoviral (Ad) vectors that do not replicate have a variety of benefits for creating HPAIV vaccines. Since their genome is stable, HPAIV antigens, expressed in vivo after vaccination, can be inserted into it. Importantly, Ad vaccines may be thermostabilized for stockpile and pandemic readiness and have an excellent safety and immunogenicity description in several human clinical studies [277].

#### 6.2.4. Universal Influenza Virus Vaccines

Lately, the formulation of a UIV has become a prominent scientific goal. Several of the positioned papers have identified major knowledge gaps, and the necessity to fund creative solutions to close these gaps was emphasized [316,317]. One approach is to enhance the variety of vaccine molecules under research, which may support in reducing the dependence on egg-based preparation and improving the breadth and durability of vaccines. Alternately, because various vaccine delivery methods elicit immune responses with varied phenotypes, different platforms might be utilized to better understand the immunological responses that are needed for widespread effectiveness and to find novel corresponds of defense. Despite being outside the purview of this review, a variety of vaccine molecules, such as conserved peptides [318], DNA [319,320], mRNA [321], nanoparticle [322,323], or VLP [324]-based vaccines, are being evaluated as UIVs that may also provide protections against newly appearing pandemics [325].

Unquestionably, improvements in creative immunogen design will make it possible to create tailored vaccination platforms that can induce both persistence and breadth of response. Examples include the employment of computationally developed immunogens such as COBRA or immunogens created using Epigraph algorithms, which might aid in boosting the amplitude over different antigens; against T cell epitopes; or against discontinuous B cell epitopes [326,327]. The creation of new HA immunogens with the goal of enhancing immune reactions against the highly preserved but immunosubdominant HA stem has made significant strides. Antibodies produced against the immunodominant and antigenically diverse HA head are often strain-specific. This is a key element in the yearly reformulation requirement for vaccinations based on the traditional IIV-based vaccines. However, the HA head domain does include highly conserved and widely neutralizing epitopes [328,329]. Even though seasonal vaccinations only sometimes generate antibodies to these epitopes and seem to do so weakly [330], head-specific monoclonal antibodies competent in neutralizing several IAV subtypes have been identified in both animals [331,332] and humans [330,333]. Most target epitopes are near the receptor binding position, and in certain instances, the monoclonal antibodies resemble the HA surface receptor sialic acid [330]. Anti-HA head monoclonal antibodies with a wide response, however, have also been discovered, and they identify epitopes different from the receptor binding site. 

The influenza vaccine industry has recently spent a lot of work attempting to understand the distinctions between the protection obtained by vaccination with various platforms and acquired through spontaneous infection. There is mounting proof that early-life exposure to the influenza virus, known as “immunological imprinting” can significantly influence later reactions and vulnerability to infection with G1 or G2 IAVs [334,335,336,337]. It is not clear if such a vaccine should provide similar immunity to G1 and G2 HAs at the same time or if vaccination should ideally occur very early in life, before the first natural infection. When creating broad-spectrum antibiotics or UIVs, this is a crucial factor to consider. These crucial issues are presently being approached by numerous cohort investigations in humans that compare communities with low vaccination scope to those with yearly seasonal influenza vaccine programs [338,339]. Significant financial assistance given in recent years to the formulation of a UIV will unquestionably have a good effect on vaccinations that stimulate protective immunity, encouraging the development of effective vaccines to guard against newly developing AIVs [340,341,342,343,344,345].

### 6.3. Challenges in the Formulation of AIV Vaccines

The significant challenge in the formulation of the vaccine against AIV is the difficulty in predicting which strains will be the reason for the next pandemic. Nevertheless, AIV of H5 and H7 subtypes are always in the spotlight. Moreover, the development of a pre-pandemic vaccine is further complicated due to the continual detection of novel AIVs. Due to the continuous evolution of the antigen and the diversity of AIVs, it is further complicated to design a vaccine [10].

Employing traditional IIV/LAIV platforms in the production of AI vaccines provides various distinct manufacturing problems in addition to low immunogenicity and safety issues. To solve this issue, repeated vaccination and an increase in doses of the vaccine are required. This will eradicate the chance of “dose-sparing” and will also lead to vaccine shortage. Some adjuvants are also needed to improve the immunogenicity of vaccines [10]. A significant problem is the lengthy production process, which makes it difficult to respond quickly to an impending epidemic. In the best-case scenario, manufacturing may be completed in 5–6 months when viable seed stocks are found and approved by the WHO in an appreciable time and when these viruses mature to adequate titers. Strain selection typically occurs 7–8 months before the influenza season [10]. However, under unforeseen conditions, the procedure might take a long time. Production could take up to 6 to 8 months. 

Another problem is the excessive dependence on the production of embryonated eggs and the possibility for major supply cutbacks if an HPAIV pandemic decimates poultry, hence reducing the availability of eggs required for manufacturing. Parallel to this, it is possible for HPAIVs and their generated vaccine seed supplies to be embryo-lethal, which makes it difficult to propagate viruses in eggs to create vaccine stocks. Furthermore, treating HPAIVs used for vaccine formulation in upgraded BSL-3 biocontainment provisions necessitates specialist personnel and practices, which raises expenses. Reverse genetic modification of HPAIVs or the utilization of non-pathogenic substitute AIVs are two options for overcoming this. Alternative platforms that are secure, flexible, elicit strong and wide protective immunity, and have manufacturing features consistent with stockpiling and pandemic preparedness would be advantageous for production.

As it is impossible to foretell the type of futuristic pandemic strain, the best approach towards designing a vaccine is the production of an inactivated vaccine that antigenically replicates the pandemic-causing IV. Vaccines of such kind aim to induce virus-neutralizing antibodies directed against HA. Nevertheless, the manufacture and production of such inactivated vaccines takes around 6 months after the identification of a pandemic-causing virus [10]. During the pandemic of 2009 caused by the A/H1N1 strain, it took very long for the preparation of the vaccine. As the result, sufficient doses of the vaccine were available in the market after the pandemic reached its peak in many countries [346]. All of these issues together highlight the requirement for new vaccines that can produce protection against distinct viruses and can also be generated rapidly at an extensive scale. The availability of these vaccines will reduce mortality and morbidity in a pandemic scenario until exactly matching vaccines become accessible. These unique vaccines ideally would produce a simultaneous protection against zoonotic influenza viruses, antigenically drifted seasonal viruses and pandemic IVs [347,348,349,350,351,352,353,354,355,356,357,358]. There have been several attempts to create an effective universal influenza vaccination that can trigger a broadly reactive response against antigenically varied viruses. In the Table 3, the recent development of vaccines against the avian influenza virus has been mentioned.

## 7. Conclusions

It is widely known that the host’s immune response to an AIV infection consists of several complex mechanisms that work in concert to play important roles in the host’s defense. Interactions between host immune mechanisms and the virus determine the virulence of the AIVs. In the case of mild infections caused by seasonal IVs, the CD8^+^ and/or CD4^+^ T cells provide considerable defense against influenza infection if these cells are already present in the host in sufficient numbers. Innate immunity is the initial line of protection against any pathogen entering the human body and also assists in the development of adaptive immunity. Adaptive immunity then clears the viral infection and promotes the host recovery. However, more severe outcomes are reported in the case of influenza infections caused by HPAIVs. Innate immunity can be inhibited due to the high replication frequency of AIVs. This can further result in the interference in the generation of the effective adaptive immunity. Inefficient or defective adaptive and/or innate immunity cannot control severe infections. Infections with the AIV are linked to a cytokine storm and a heightened innate immunological response. When compared to seasonal IVs, the antibody response to the HA is less potent. Multiple immunological effectors, mostly CD8^+^ T cells, promote the recovery from a severe illness. The formulation of pandemic influenza vaccines has come a long way, particularly with the addition of oil-in-water adjuvants and the prime-boost strategy, which combines a variety of molecules, such as DNA, LAIV or a vector vaccine succeeded by a protein vaccine. These methods expand the antibody response from the extremely strain-specific response observed with an unadjuvanted subunit vaccination. While there has been a significant improvement in our understanding of the AIV–host interaction, more research is still needed to fully grasp the dynamics of the host immune system upon recognition of the evolving AIVs. The creation of new antiviral drugs and the development of the improved vaccinations and immunization methodologies will be made possible by closing these gaps.

Furthermore, challenges of the low immunogenicity of vaccines against AIV and AIV antigenic diversity can be overcome by developing a novel vaccine that has a broad reactivity against different AIV subtypes. The generation of a novel vaccine is one of the crucial steps for the prevention of a pandemic-like scenario. Immune responses produced upon vaccination can be assessed by obtaining the human sera from clinical investigations and evaluating it against the chosen panel of viruses. There is a critical necessity to generate antibodies and innovative tools to evaluate the immunology of ferrets, as ferrets are considered to be the “gold standard” of the influenza animal model. The creation of vaccines that produce wide cross-reactivity against all IAVs within a subtype or a UIV that gives protection against all IAV subtypes, which are the intermediate and ultimate aims of vaccine research, are both within reach. To overcome the challenges in preparing a pandemic vaccine, various vaccine platforms should be combined so that a multivalent pandemic vaccine can be developed that will elicit both B and T cell responses. For developing an efficient pandemic vaccine against AIV, efforts for combining both sorts of immune responses will be crucial.

## Figures and Tables

**Table 3:** Recent development of vaccines against avian influenza virus.

S. No.	Name of Vaccine	Type of Vaccine	Immune Response	Virus Subtype	Reference
1	H5-Re13, H5-Re-14, H7-Re-4	Inactivated virus vaccine	Antibody-mediated response	H5N1, H5N6, H5N8, H7N9	[348]
2	H5 candidate vaccine strain A/17/turkey/Turkey/05/133 H5N2	Live-attenuated influenza virus	Antibody-mediated response	H5N2	[303]
3	Nobilis Influenza H5N2 vaccine	Inactivated virus vaccine	Antibody-mediated response	H5N2	[349]
4	MF59-adjuvanted seasonal influenza vaccine (Fluad^®^) Novartis Vaccines and Diagnostics Inc., MA, USA	Trivalent inactivated vaccine	Antibody, Cell-mediated response	H5N1	[350]
5	AS03-adjuvanted prepandemic H5N1 influenza vaccine	Inactivated virus vaccine	Antibody and cell- mediated response	H5N1	[351]
6	H7N9 LAIV	Live-attenuated influenza virus	Antibody-mediated response	N7N9	[352]
7	Beta-propriolactone whole-inactivated virus	Inactivated virus vaccine	Antibody and cell-mediated responses	H9N2	[353]
8	H5N1 pandemic live-attenuated influenza virus vaccination	Live-attenuated influenza virus	T-cell-mediated response	H5N1	[310]
9	Newcastle Disease Virus H5 vaccine	Vector-based vaccine	Antibody, mucosal and cell-mediated response	H5N1	[354]
10	H5N1 influenza virus vaccine (Manufactured by: Sanofi Pasteur, Inc.)	Inactivated monovalent influenza virus vaccine	Antibody-mediated response	H5N1	[355]
11	Pandemic influenza vaccine H5N1 Astrazeneca	Live-attenuated influenza virus	Antibody-mediated response	H5N1	[356]
12	H7 pandemic live-attenuated influenza vaccines (pLAIV)	Live-attenuated influenza virus	Antibody-mediated response	H7N7	[357]
13	H9N2 avian influenza virus-like particle vaccine	Virus-like particle vaccine	Antibody and cell-mediated responses	H9N2	[358]

## Data Availability

Not applicable.

## Supplementary Materials

The following supporting information can be downloaded at: https://www.mdpi.com/article/10.3390/vaccines11030593/s1.
