# Supplementary material for: Immune Control of Avian Influenza Virus Infection and Its Vaccine Development
Source: Vaccines (Basel). 2023 Mar 4;11(3):593. doi: 10.3390/vaccines11030593 (PMC10058720; doi:10.3390/vaccines11030593)
Supplement: Supplementary file 1 [file vaccines-11-00593-s001.zip › vaccines-2153184-supplementary.pdf]

SUPPLEMENTARY FILE

1. Epidemiology

Different individuals may experience a variety of pathological conditions after contracting the H1N1 virus. This can result in incomprehensible flu-like symptoms or acute pneumonia. The most popular approach for preventing the disease is H1N1 swine flu vaccination. The Food and Drug Administration (FDA) has sanctioned a swine flu vaccine in September 2009, and various tests conducted by the National Institutes of Health (NIH) reports that within 10 days of administration, one dosage of vaccine will produce enough antibodies to fight against the virus [1].

These viruses first appeared in China and have since spread to many other nations, including Egypt, India, Bangladesh, Vietnam, and Indonesia, where they are endemic in chicken. Evolution by drift as well as reassortment has led to the diversification of this GsGd lineage into 10 clades plus high-order subclades. Since 2014 to present, Egypt and Indonesia got predominantly hit by this HPAIV [2].

Most H5N1 infections have been reported in Egypt, Indonesia, Vietnam, Cambodia, and China, with total cases ranging from 52 to 344 [3]. Low pathogenic avian H5N6 IV epidemics in hens were common in China, Vietnam, and Laos in 2014 and 2015. Two instances of H5N6 in humans have recently been reported from China, and the clinical symptoms were determined to be acute [3][4]. China is the only country to have documented a severe human illness caused by the H5N6 AIV subtype, with 19 cases, 13 of which resulted in death [5]. The mortality rate for H5N1 infections is above 50%. H5N1 infection rates are anticipated to be larger than 30%, even though multiple research efforts have concentrated on estimating more realistic rates, considering the mild asymptomatic cases and limited transmissibility [6]. The first case of the human H6N1 influenza virus was documented on May 20, 2013 in Taiwan [7].

H7N9 virus is most frequently discovered in live chicken markets across China and only occasionally on poultry farms. Majority of the patients with H7 virus infection shows mild-influenza like symptoms though one fatal case of H7N7 infection has also been reported. On the other hand, H7N9 virus primarily infects elderly people and has high risk fatality risk. H7N9 viruses cause severe illness in addition to increased risk of ARDS and pneumonia [8]. Even though the H7N9 virus causes serious human illnesses with a 98% hospitalization rate, it is an LPAIV affecting birds. [8] For the H7N9 virus, the human incubation period ranges from 1–10 days, with a mean of 5 days [9].

H7N2 is an LPAIV but has the potential to be converted to an HPAIV form by undergoing mutations at the HA cleavage site [10]. No human influenza case with the H7N2 virus was reported before the 2002 outbreak in Virginia [11]. Because of their improved infectivity and ability to resist selective pressure through antigenic alteration, H9N2 viruses of genotype 57 (G57) are particularly advantageous in chickens. Between 2010 and 2013, widespread chicken H9N2 outbreaks in China were caused by G57 AIVs [12].

It is noteworthy that the core genes for both H7N9 and H10N8 were derived from the H9N2 virus, a persistent reassortment with local poultry H9N2 strains assumed to have an elevated potential for epidemic or pandemic [13]. Nevertheless, it is not exactly clear how these H9N2 chicken viruses promoted the emergence of the recently discovered H7N9 virus [12].

2. Pathogenesis of AIV illness in gallinaceous birds

A cleaved HA is furthermore needed for the fusion of the viral envelop with the membrane of the endosome. Proteases similar to trypsin found in the mucosal epithelial cells of the respiratory and gastrointestinal tracts and furin-like proteases found in several organs are responsible for the breakdown of the HA protein of LPAIVs and HPAIVs, respectively [14][15][16].

There are two different types of HPAIV disease pathogenesis, depending on how the virus prefers to replicate in endothelial cells. In the initial form, some HPAI H5N1 virus strains reproduce in endothelial cells, leading to blood vessel injury. Edema, haemorrhage, and microthrombosis are caused by this vascular injury, which leads to rapid mortality and multiple organ failure. In the second type, viral multiplication occurs extensively in parenchymal cells of several organs but not in endothelial cells, leading to acute necrosis, organ failure, inflammation, and rapid death [17][18][19].

There are three distinct pathophysiological pathways by which AIVs produce significant cell damage and cell death, according to Pantin-Jackwood and Swayne: (i) Direct viral replication in cells, (ii) indirect effects from the generation of cytokines and (iii) ischemic consequences due to viral multiplication in endothelial cells, which cause acute organ damage and host death [17]. Nonetheless, it is still unclear which molecular processes cause cell damage and death, and whether they result from a single mechanism or a mixture of several different mechanisms.

3. Clinical features of severe disease

3.1. Clinical findings of H5N1 infection

An investigation recorded the epidemiologic, clinical, and radiologic characteristics of all confirmed instances of H5N1 virus infection in patients admitted to Van, Turkey's Yuzuncu Yil University Hospital between the period of December 31, 2005, and January 10, 2006. Eight patients were found to have H5N1 virus infection. Besides, eight patients, ranging in age from 5 to 15, shared a history of intimate contact with sick or diseased chickens. The average duration from exposure to the start of the illness was 5.0 ± 1.3 days. All the patients presented with fever. Seven of the patients had clinical and radiologic pneumonia symptoms, and four succumbed to illness. The diagnosis was made using a polymerase chain reaction (PCR) assay after enzyme-linked immunosorbent assay (ELISA), and a rapid influenza test for each patient came out to be negative [20].

3.2. Clinical findings in H7N9 infection

An investigation collected and evaluated clinical, epidemiologic, radiologic, and virologic reports from three patients admitted to Shanghai Ruijin Hospital who had been diagnosed with A H7N9 infection between March 1 and April 30, 2013. Two patients were either directly or indirectly exposed to chicken in the food marketplace. They first showed signs of fever and fast-developing pneumonia that did not improve with antibiotic treatment. The number of days between the beginning of symptoms and the initiation of respiratory failure ranged from 7 to 11. Invasive secondary bacterial infections were found in two cases. Following the onset of symptoms, both patients died between days 7 and 86 [21]. The finding specified that the novel reassortant avian influenza H7N9 virus could cause ARDS in people when it is transmitted from cross-species birds to person.

Furthermore, a study by Chen and colleagues found four patients (average age 56 years), all of whom had contact with poultry three to eight days before the beginning of illness. They first showed signs of fever and fast-developing pneumonia that did not improve

with antibiotic treatment. Leucopenia and lymphopenia, impaired liver activity, markedly raised blood cytokine or chemokine levels, and intravascular coagulation were all symptoms of advanced illness, and two patients were reported to have succumbed to disease. Compared to samples from throat swabs, sputum samples had a higher likelihood of testing positive for the H7N9 virus. The patient's viral isolation closely matched one from a market chicken with which it was associated epidemiologicall [22]. The finding emphasized that improvements in illness management, epidemic control, and pandemic preparation would result from monitoring viral evolution and additional research into disease pathogenesis.
The increased mortality may be linked to advanced age, obesity and the existence of comorbid conditions. In advanced stages of the illness, pulmonary fibrosis might be observed.

3.3. Clinical findings in H5N6 infection

Another investigation looked at 5 new H5N6 cases that appeared in Sichuan Province, China, between May 2021 and July 2021. All five individuals that were examined during the study reported having contact with live chickens before developing their illnesses. The symptoms reported by the patients included headache, asthma, nasal stuffiness, cough and fever. The genomic study of H5N6 genomes revealed the characteristics of AIV genomic reassortments and significant residue substitutions [23].

4. Diagnosis of avian influenza virus

RT-PCR is the traditional method for quickly and accurately diagnosing and typing RNA viruses, including IAVs [24]. The wide scale use of PCR-based assays or assay components has also opened up the possibility of expanding distribution and standardizing procedures both within and between nations [25][26][27]. The PCR-based assays have the advantage of quick identification (up to an hour) and analytical detection limits of 5–100 viral particles, which usually achieve or surpass the anticipated virus recovery limits for traditional virus isolation approaches [28].

In recent years, sequence investigation, whether standard Sanger or cycle-sequencing or substitutes like rapid high-throughput pyrosequencing, has also been utilized for virus diagnosis with exceptionally high phylogenetic resolution in addition to epidemiological research. Full-genome sequence analysis is making steady progress toward real-time and routine use due to advancements in microfluidics and computer-assisted analysis tools [29][30].

As a new substitute for nucleic acid targeting, a prototype IV glycan microarray was utilized to screen for IV receptor specificity. This single chip had hundreds of different glycan structures. Receptor-binding tests for diagnostic purposes are not yet well established, despite the fact that receptor particularity is connected to virulence and trans-species transmittance, but they may offer a potential new avenue for influenza and other comparable viruses. Microarray technology, which was once thought to be too expensive for use in routine diagnostics, is now being formulated especially for cost-friendliness. In the not-too-distant future, it might be sold at prices comparable to those of other molecular-based diagnostics [31].

Lyophilized constituents, multiple kit patterns and several equipment platforms for RNA recovery, as well as fluorescence detection, have been used for the diagnosis of AIV and the distinct identification of the AI H5 subtype. High throughput formats and robotics have been used in different parts of the PCR procedure, from the nucleic acid isolation to PCR cycling, to offer a surge capability for the laboratory workload in the event of a possible AI upsurge and the related public health response attempt [28].

5. Immune response to Avian influenza viruses

5.1. Innate immune response

The NS1 expressed by the viral genome has been reported to suppress IFNs-α and IFN-β induction. A study demonstrated how avian H5N1 NS1 production in HeLa cells prohibits IFN signaling. H5N1 NS1 was found to suppress the nuclear translocation of the phospho-STAT2 and the formation of the IFN-inducible STAT1:1-, STAT1:3- and STAT3:3-DNA complexes; and lowered the tyrosine phosphorylation of STAT1, STAT2 and STAT3 upon IFN stimulation. The inhibition of the IFN-inducible STAT signaling by NS1 in HeLa cells was demonstrated to be the result of the NS1-mediated repression of the IFN receptor component, IFNAR1. The research demonstrated that NS1 can straightly impede with IFN signaling to increase viral multiplication, but IFN therapy can still overcome these restrictive results to stop the spread of the H5N1 and H1N1 viruses [32].

When AIVs engage with the dendritic cells (DCs) that line the URT, the adaptive immune response is initiated [33]. Primary or secondary immunological reactions start when DCs move to nearby lymph nodes. IAV proteins carry peptides attached to MHC class I and class II proteins to the cytoplasmic and lysosomal processing pathways of the infected cell, where they are identified by $CD8^{+}$ CTLs and $CD4^{+}$ T helper (Th) cells, correspondingly. When AIVs infect DCs, they often do not cause cell death or viral infection, whereas HPAIVs such as H5N1 can do both and can also eliminate select subsets of human DCs [34].

Lately, matrix Metalloprotease (MMP) 9 has been demonstrated to mediate excessive neutrophil movement into the respiratory tract in response to IV multiplication. MMPs are crucial for immunity as a result, and their proteolytic function, which blocks cytokine and chemokine activity, may reduce the likelihood of inflammation. However, extreme responses, such those brought on by an HPAIV infection, might result in pathology [35].

5.2. Adaptive immune response

The recognition of IV is essential for not only initiating the adaptive immune response against the invading virus but also to limit the inflammatory responses at the initial stages of infection and viral replication. Activation of inflammasomes in the lungs and IL-1R signaling in pulmonary DCs are also critical following an influenza infection for the generation of adaptive immune responses against the virus, as the result of which antigen-captured DCs migrate towards the draining mediastinal lymph nodes. If the gut microbiota is healthy, it can provide tonic IFN signals that result in the steady-state mRNA production of pro-IL-18 and pro-IL-1 in the lungs. Some studies have been conducted that highlight the beneficial effects of microbiota-derived metabolites in antiviral immunity or against the influenza virus infection [36].

In another report, in comparison to H5N1 and H7N9 patients, H5N6 patients were demonstrated to have greater rates of lymphopenia, as well as raised alanine aminotransferase and LDHA levels. When compared to individuals infected with other AIV subtypes, hypercytokinemia was observed in H5N6 patients at a noticeably greater frequency. When adaptive immunity was examined, it was discovered that the H5N6-infected survivor had both humoral and cellular responses, but cellular responses were not present in the people who died due to illness. Additionally, compared to those who died, the surviving patient demonstrated reduced levels of both pro- and anti-inflammatory cytokines and chemokines [5].

Furthermore, the ability of T cells other than the $CD8^+$ T cell subsets to protect against the avian IAV infection has also been suggested [37]. Adults' healthy $CD4^+$ T cells specifically displayed a cross-reactivity with H5N1 viruses. Since cross-reactive $CD4^+$ T cells are protective against illness with human IAVs, such cross-reactivity is advantageous during an AIV illness [38][39]. In addition, acute illness with the avian H7N9 virus has been linked with the depletion of the innate mucosal-associated invariant T (MAIT) cell fraction from the blood. Since viruses frequently cause the MAIT cells to become activated, this might result in the up-regulation of cytotoxic molecules and the production of antiviral cytokines , which may hasten the healing process. However, it is unclear how exactly MAIT cells contribute to avian IAV infection [40].

5.3. Distinct Host Immunological Responses to LPAIV and HPAIV in Birds

The host immunological responses and course of the illness in birds infected with AIVs are influenced by the virus strains, species and immunological condition of the birds. The main determinants of the age-dependent vulnerability to the influenza infection may not be cellular host components engaged in viral multiplication but rather the potency of host responses at the transcriptional level [41][42]. A few research studies have looked at how birds' immune systems react to an LPAIV infection. IFNs, pro-inflammatory cytokines and chemokines, and other immune-associated genes, are either hardly activated or unaffected by an LPAIV infection [43][44]. In contrast, inflammatory cytokines (TGFB3, IFNA and IFNB) and TLRs are effectively induced in several tissues by the LPAIV H9N2 infection in chickens [45]. Additionally, chickens activate several transcription factors, including c-Jun, IRF-3 and p50, as well as the mitogen-activated protein kinase (MAPK)/extracellular signal-regulated kinases (ERK) signaling pathways, which are crucial for viral replication, the generation of pro-inflammatory cytokines and the apoptotic reactions to an LPAIV infection [46][47]. It is possible that the variations in viral multiplication and the ensuing host reactions in important organs are the cause of the infectivity and lethality discrepancies between HPAIV and LPAIV illness in birds [48]. Significantly, HPAIV-infected chickens and ducks demonstrated greater levels of expression of RIG-I, IL-6, IL-8, IFNs, ISG12-2, interferon-induced protein with tetratricopeptide repeats (IFIT5), MHC-I, IFN-induced transmembrane protein 1 (IFITM1) and other genes, while little to no response was reported in the case of LPAIV-infected chickens and ducks [42][49][50]. One hypothesis is that the NS1 protein of LPAIVs more effectively blocks the viral stimulation of the IFN pathway than the NS1 of HPAIV [51]. Another study contends that one of the factors contributing to the increased infectivity of H5N1 viruses in chickens may be a reduction in the activation of NK cells [52]. In addition, according to another study, the greater pathogenicity of HPAIVs may be linked to the virus's effective and quick reproduction, and prompts strong host immune responses [48][43]. Conducting more research studies is necessary to determine which viral and host variables regulate the gene expression that makes birds vulnerable or resistant to AIV illnesses. Such information will greatly benefit the development of specific treatment approaches depending on the level of viral pathogenicity.

5. Vaccine Development

A vaccination that triggers a cell-mediated immunological response may be helpful in at least two situations in the case of a pandemic. First, even while a cell-mediated immunological reaction may not be able to stop an individual from becoming infected, it can help protect them from serious disease and even death due to influenza. For a pandemic influenza

vaccination, this result could be tolerable even if it might not be viable for a seasonal influenza vaccine. Second, vaccinations that induce defense through a cell-mediated immune response may be beneficial if the incubation period and duration of infection with AIVs are greater than those with human IVs [53].

Even during the pandemic of 2009, individuals possessing an appreciable level of already-existing $CD8^+$ T-cells which are specific for AIV, were less prone to acquire acute influenza illness after infection [54].

Numerous of these substitute molecules are appealing because they enable the simultaneous administration of multiple conserved antigens or epitopes, some of them have intrinsic immunostimulatory or intrinsic "adjuvanting" properties that could enhance amplitude, some molecules present antigen in new arrangements like redundant particulate formulations, and others can be quickly modified and scaled up in response to pandemics. Many of these requirements are specifically met by vaccinations with viral vectors. They can activate immunostimulatory pathways by entering cells and delivering their nucleic acid genome, and once the transgenic is produced, this milieu may promote improvements in immunological potency [55].

The fact that the interior proteins of the virus that are the objectives of the cell-mediated immunological response tend to be conserved and are less vulnerable to genetic drift than the antibody-binding positions on the HA and NA glycoproteins is one possible benefit of a vaccine that prompts a cell-mediated immune response over one that stimulates a defensive antibody reaction. [53].

Virus-specific T cells cannot confer sterile immunity but play a notable part in providing clinical protection against the zoonotic influenza virus, pandemic influenza virus and antigenically drifted seasonal viruses. Inactivated vaccines inefficiently induce responses of $CD8^+$ T cells [56]. For inducing the virus-particular $CD8^+$ T cells efficiently, a vaccine delivery system that permits the production of viral de novo protein synthesis in the human body is required.

Split-virion vaccines, also known as sub-unit vaccines, are WIV vaccines that have gone through further purification using detergents to produce membrane elements containing both HA and NA (split-virion), or nearly exclusively HA-based immunogens (sub-unit) [57].

Appreciable levels of HA-particular $CD4^+$ and $CD8^+$ T cells are induced by the rMVA-H5 vaccine in mice [54]. These T cells have been reported to target the epitopes that are conserved by different HA clades. rMVA allows for the expression of multiple antigens, which can be easily modified to include optimal cross-reactive immune responses. Protective action against the homologous and heterologous challenge has also been observed in mice, when vaccinated with an rMVA vaccine expressing an integration of HA and NA, or the HA and NP of an A/H5N1 virus [58].

Consequently, the preserved HA head epitopes could be a new target for the development of IV vaccines in the future. Alternative vaccination platforms and the application of structural biology approaches may offer more information and allow for a better induction of immune responses against these uncommon or occluded epitopes than is possible with traditional vaccine platforms [59][60].

5.1. Clinical vaccine development against avian influenza virus

In two multicentre, parallel-assignment, randomized studies, conducted in Australia in 2008, healthy adult volunteers were given either two doses of the H5 HA vaccine plus/minus aluminium phosphate ($AlPO_4$) adjuvant (phase I trial) or two doses of the H5 HA vaccine plus/minus $AlPO_4$ adjuvant (phase II trial) (NCT00136331, NCT00320346). Six months

following dosage 2, a booster dose of immunization was made available (phase I trial only). No unforeseen major adverse effects were observed, and all formulations were well-tolerated. The strongest immune responses were induced by two doses of formulations containing 30 µg or 45 µg H5 HA adjuvanted, with microutilisation (MN) antibodies persisting for up to six months after vaccination. The immunogenicity of the 7.5 and 15 µg formulations (+/- adjuvant) was less than that of the higher dosage formulations; after 6 months, hemagglutination-inhibition (HAI) and MN antibody titres had nearly reached pre-vaccination levels, but had returned to post-dose 2 levels following the booster dose. Immunological responses from the phase I study showed moderate levels of cross-protective MN antibodies against two distinct, presently perpetuating clade 2 H5N1 viruses [61].

Furthermore, a phase 1 and 2 randomised, dose-escalation investigation with six subgroups accessed the safety of an H5N1 whole-virus vaccine generated on Vero cell cultures and examined its capacity to create antibodies that could neutralize various H5N1 strains (NCT00349141). In two visits separated by 21 days, 275 persons between the ages of 18 and 45 got two doses of the vaccine, each of which included 3.75, 7.5, 15, or 30 µg of HA antigen with an alum adjuvant or 7.5 or 15 µg of HA antigen without an adjuvant. The first, day 21, and day 42 of the experiment involved serologic testing. In addition to the clade 1 virus strain, the immunization also produced a neutralizing immunological reaction against the clade 2 and 3 viruses. Adjuvant use had no positive impact on the antibody response. With formulations comprising 7.5 and 15 µg of HA antigen without adjuvant, the highest responses to the vaccination strain were achieved. The most frequent unfavorable effects for all vaccination formulations were slight discomfort at the injection site and headache [62].

A potential H5N1 vaccination with MF59 adjuvant received regulatory clearance thanks in large part to a critical trial conducted in 2010. Healthy volunteers between the ages of 18 and 60 were given one of four randomized regimens with a 1-, 2-, 3-, or 6-week gap between the two doses of the vaccine. According to the single radial hemolysis (SRH) study, the H5N1/turkey/Turkey/1/05) influenza antigen elicited a heterologous immunological response in the proposed vaccine. The vaccine met two of the three European licensing criteria with seroconversion frequencies of 69% and 65% in the groups allocated to a 2-week delay between doses, correspondingly. There were no significant unfavorable reactions following immunization. Mild to severe injection site discomfort and fatigue were the adverse reactions that were most often reported within 7 days after receiving the initial and second dosages of the vaccine [63]. In the healthy volunteers, two 7.5 µg doses of the H5N1 influenza vaccine given 2, 3, or 6 weeks apart provided H5N1-specific immunity and satisfied the Committee for medicinal Products for Human Use (CHMP) licencing requirement for seroprotection. When the 2 vaccination doses were given either 2 or 3 weeks apart, clinically significant amounts of heterologous immunity were seen; nevertheless, the licencing requirement for seroprotection was not reached in this instance.

One hundred twenty-five young, healthy people were randomly assigned to get two intramuscular inoculations of either a placebo or 7.5, 15, 45, or 90 µg of HA of an inactivated subunit influenza A (H7N7) vaccination, four weeks apart, on each occasion (NCT00546585). The evaluations of reactogenicity showed that the vaccines were well tolerated. In tests conducted at a central laboratory, only one participant showed an increase in neutralizing antibodies with a final titer of more than equal to 1:40 and a more than equal to 4-times increase in serum HAI antibody response four weeks after dosage two. Four out of the five received either 45 or 90 µg of HA. Only 36% of the 90 µg HA group had a 4-increase increase in antibody in this test, and only one of them attained a titer of more than equal to 1:32, according to a more sensitive HAI testing performed at the research site. It was concluded that although safe, this inactivated influenza A vaccination had a weak immunogenic response in humans [64].

In phase I clinical trials (NCT00755703 and NCT01806909), the efficacy of intranasal delivery of the Ad5 and Ad4 influenza vectors was evaluated in light of the deficient humoral immunity following oral administration of either the Ad5- or Ad4-based vaccines and the possibility that the route of vaccine delivery may have adversely affected the elicitation of strong humoral immunogenicity. Although formal published results have not yet been disclosed, these trials together sought to assess protection and immunogenicity in healthy people from ages 18 to 49. Ad4-H5-VTN), a replication-competent PaxVax vaccine, was investigated in a more latest clinical investigation in humans (NCT01443936) using the tonsil, intranasal or oral routes (238). 56 healthy people between the ages of 18 and 49 received this vaccination in doses ranging from 10^3 and 10^8 viral particles via the tonsillar or intranasal route and 10^{10} viral particles by oral administration [65]. The tonsillar and intranasal groups both experienced Ad4 seroconversion at doses more than 104 virus particles. However, peripheral memory B cell populations included antibodies with a wide range of neutralizing activity and potency, despite the restricted total serum neutralization by MN test against H5. Importantly, even though this was not revealed at the serum amount, immunization with the replication-competent Ad4-H5 vector led to extended rises in somatic hypermutation (SHM) and, as a result, improved antibody efficacy against H5 for numerous months.

Furthermore, the protection and immunogenicity of the IVACFLU-A/H5N1 vaccine were being evaluated in a Phase 2/3 double-blinded, randomized trial in normal individuals (NCT02612909). In phase two trial, 300 individuals were arbitrarily allocated to one of three groups for a dosage selection research (15 mcg, 30 mcg, or placebo). All individuals' protection and immunogenicity were evaluated. In Phase 3, 630 individuals were arbitrarily allocated to get either the placebo (n = 105) or the 15 mcg dosage of the IVACFLU-A/H5N1 vaccination chosen in Phase 2. All Phase 3 participants had their safety evaluated, and a selection of subjects had their immunogenicity tested. The majority of the adverse effects were modest and transient, and the vaccination was well tolerated. 60 percent of participants in the vaccination group in Phase 3 reported experiencing mild discomfort at the injection site as the most frequent side event. Both the 15 mcg and 30 mcg dosages were immunogenic in Phase 2, therefore Phase 3 testing focused on the lower dose. HAI seroconversion rates were 68 percent overall in Phase 3, MN seroconversion rates were 51 percent, and SRH seroconversion rates were 56 percent [66].

6. References

1. Yang, R.; Sun, H.; Gao, F.; Luo, K.; Huang, Z.; Tong, Q.; Song, H.; Han, Q.; Liu, J.; Lan, Y.; et al. Human Infection of Avian Influenza A {H3N8} Virus and the Viral Origins: A Descriptive Study. *Lancet Microbe* **2022**, *3*, e824--e834.

2. Sutton, T.C. The Pandemic Threat of Emerging H5 and H7 Avian Influenza Viruses. *Viruses* 2018.

3. Su, S.; Bi, Y.; Wong, G.; Gray, G.C.; Gao, G.F.; Li, S. Epidemiology, Evolution, and Recent Outbreaks of Avian Influenza Virus in China. *J. Virol.* **2015**, doi:10.1128/jvi.01034-15.

4. Yang, Z.-F.; Mok, C.K.P.; Peiris, J.S.M.; Zhong, N.-S. Human Infection with a Novel Avian Influenza A(H5N6) Virus. *N. Engl. J. Med.* **2015**, doi:10.1056/nejmc1502983.

5. Bi, Y.; Tan, S.; Yang, Y.; Wong, G.; Zhao, M.; Zhang, Q.; Wang, Q.; Zhao, X.; Li, L.; Yuan, J.; et al. Clinical and Immunological Characteristics of Human Infections With

H5N6 Avian Influenza Virus. *Clin. Infect. Dis.* **2019**, doi:10.1093/cid/ciy681.

6. Li, F.C.K.; Choi, B.C.K.; Sly, T.; Pak, A.W.P. Finding the Real Case-Fatality Rate of H5N1 Avian Influenza. *J. Epidemiol. Community Health* **2008**, doi:10.1136/jech.2007.064030.

7. Wu, H.; Yang, F.; Liu, F.; Lu, R.; Peng, X.; Chen, B.; Yao, H.; Wu, N. Isolation and Characterization of Novel Reassortant H6N1 Avian Influenza Viruses from Chickens in Eastern China. *Virol. J.* **2018**, doi:10.1186/s12985-018-1063-y.

8. Zhao, M.; Chen, J.; Tan, S.; Dong, T.; Jiang, H.; Zheng, J.; Quan, C.; Liao, Q.; Zhang, H.; Wang, X.; et al. Prolonged Evolution of Virus-Specific Memory T Cell Immunity after Severe Avian Influenza A (H7N9) Virus Infection. *J. Virol.* **2018**, doi:10.1128/jvi.01024-18.

9. WHO Avian and Other Zoonotic Influenza. *Who* **2018**.

10. Sekine, W.; Takenaka-Uema, A.; Kamiki, H.; Ishida, H.; Matsugo, H.; Murakami, S.; Horimoto, T. Adaptation of the H7N2 Feline Influenza Virus to Human Respiratory Cell Culture. *Viruses* 2022, *14*.

11. Terebuh, P.; Adija, A.; Edwards, L.; Rowe, T.; Jenkins, S.; Kleene, J.; Fukuda, K.; Katz, J.M.; Bridges, C.B. Human Infection with Avian Influenza A(H7N2) Virus—Virginia, 2002. *Influenza Other Respi. Viruses* **2018**, doi:10.1111/irv.12546.

12. Pu, J.; Wang, S.; Yin, Y.; Zhang, G.; Carter, R.A.; Wang, J.; Xu, G.; Sun, H.; Wang, M.; Wen, C.; et al. Evolution of the H9N2 Influenza Genotype That Facilitated the Genesis of the Novel H7N9 Virus. *Proc. Natl. Acad. Sci. U. S. A.* **2015**, doi:10.1073/pnas.1422456112.

13. Ma, C.; Lam, T.T.-Y.; Chai, Y.; Wang, J.; Fan, X.; Hong, W.; Zhang, Y.; Li, L.; Liu, Y.; Smith, D.K.; et al. Emergence and Evolution of H10 Subtype Influenza Viruses in Poultry in China. *J. Virol.* **2015**, doi:10.1128/jvi.03167-14.

14. Klenk, H.D.; Rott, R.; Orlich, M.; Blödorn, J. Activation of Influenza A Viruses by Trypsin Treatment. *Virology* **1975**, doi:10.1016/0042-6822(75)90284-6.

15. Meseko, C.A.; Oluwayelu, D.O. Avian Influenza. In *Transboundary Animal Diseases in Sahelian Africa and Connected Regions*; 2019 ISBN 9783030253851.

16. Stieneke-Grober, A.; Vey, M.; Angliker, H.; Shaw, E.; Thomas, G.; Roberts, C.; Klenk, H.D.; Garten, W. Influenza Virus Hemagglutinin with Multibasic Cleavage Site Is Activated by Furin, a Subtilisin-like Endoprotease. *EMBO J.* **1992**, doi:10.1002/j.1460-2075.1992.tb05305.x.

17. Pantin-Jackwood, M.J.; Swayne, D.E. Pathogenesis and Pathobiology of Avian Influenza Virus Infection in Birds. *OIE Rev. Sci. Tech.* **2009**, doi:10.20506/rst.28.1.1869.

18. Swayne, D.E. Understanding the Complex Pathobiology of High Pathogenicity Avian Influenza Viruses in Birds. In Proceedings of the Avian Diseases; 2007.

19. Kuiken, T.; van den Brand, J.; van Riel, D.; Pantin-Jackwood, M.; Swayne, D.E. Comparative Pathology of Select Agent Influenza a Virus Infections. *Vet. Pathol.*

2010, doi:10.1177/0300985810378651.

20. Oner, A.F.; Bay, A.; Arslan, S.; Akdeniz, H.; Sahin, H.A.; Cesur, Y.; Epcacan, S.; Yilmaz, N.; Deger, I.; Kizilyildiz, B.; et al. Avian Influenza A (H5N1) Infection in Eastern Turkey in 2006. *N. Engl. J. Med.* **2006**, doi:10.1056/nejmoa060601.

21. Wang, X.F.; Shi, G.C.; Wan, H.Y.; Hang, S.G.; Chen, H.; Chen, W.; Qu, H.P.; Han, B.H.; Zhou, M. Clinical Features of Three Avian Influenza H7N9 Virus-Infected Patients in Shanghai. *Clin. Respir. J.* **2014**, doi:10.1111/crj.12087.

22. Chen, Y.; Liang, W.; Yang, S.; Wu, N.; Gao, H.; Sheng, J.; Yao, H.; Wo, J.; Fang, Q.; Cui, D.; et al. Human Infections with the Emerging Avian Influenza A H7N9 Virus from Wet Market Poultry: Clinical Analysis and Characterisation of Viral Genome. *Lancet* **2013**, doi:10.1016/S0140-6736(13)60903-4.

23. Xiao, C.; Xu, J.; Lan, Y.; Huang, Z.; Zhou, L.; Guo, Y.; Li, X.; Yang, L.; Gao, G.F.; Wang, D.; et al. Five Independent Cases of Human Infection with Avian Influenza H5N6 - Sichuan Province, China, 2021. *China CDC Wkly.* **2021**, *3*, 751–756, doi:10.46234/ccdcw2021.187.

24. Herrmann, B.; Larsson, C.; Zweygberg, B.W. Simultaneous Detection and Typing of Influenza Viruses A and B by a Nested Reverse Transcription-PCR: Comparison to Virus Isolation and Antigen Detection by Immunofluorescence and Optical Immunoassay (FLU OIA). *J. Clin. Microbiol.* **2001**, doi:10.1128/JCM.39.1.134-138.2001.

25. Wright, K.E.; Wilson, G.A.R.; Novosad, D.; Dimock, C.; Tan, D.; Weber, J.M. Typing and Subtyping of Influenza Viruses in Clinical Samples by PCR. *J. Clin. Microbiol.* **1995**, doi:10.1128/jcm.33.5.1180-1184.1995.

26. Spackman, E.; Senne, D.A.; Myers, T.J.; Bulaga, L.L.; Garber, L.P.; Perdue, M.L.; Lohman, K.; Daum, L.T.; Suarez, D.L. Development of a Real-Time Reverse Transcriptase PCR Assay for Type A Influenza Virus and the Avian H5 and H7 Hemagglutinin Subtypes. *J. Clin. Microbiol.* **2002**, doi:10.1128/JCM.40.9.3256-3260.2002.

27. Lau, L.T.; Banks, J.; Aherne, R.; Brown, I.H.; Dillon, N.; Collins, R.A.; Chan, K.Y.; Fung, Y.W.W.; Xing, J.; Yu, A.C.H. Nucleic Acid Sequence-Based Amplification Methods to Detect Avian Influenza Virus. *Biochem. Biophys. Res. Commun.* **2004**, doi:10.1016/j.bbrc.2003.11.131.

28. Das, A.; Spackman, E.; Senne, D.; Pedersen, J.; Suarez, D.L. Development of an Internal Positive Control for Rapid Diagnosis of Avian Influenza Virus Infections by Real-Time Reverse Transcription-PCR with Lyophilized Reagents. *J. Clin. Microbiol.* **2006**, doi:10.1128/JCM.00639-06.

29. Obenauar, J.C.; Denson, J.; Mehta, P.K.; Su, X.; Mukatira, S.; Finkelstein, D.B.; Xu, X.; Wang, J.; Ma, J.; Fan, Y.; et al. Large-Scale Sequence Analysis of Avian Influenza Isolates. *Science (80-.).* **2006**, doi:10.1126/science.1121586.

30. Pourmand, N.; Diamond, L.; Garten, R.; Erickson, J.P.; Kumm, J.; Donis, R.O.; Davis, R.W. Rapid and Highly Informative Diagnostic Assay for H5N1 Influenza Viruses. *PLoS One* **2006**, doi:10.1371/journal.pone.0000095.

31. Stevens, J.; Blixt, O.; Glaser, L.; Taubenberger, J.K.; Palese, P.; Paulson, J.C.; Wilson, I.A. Glycan Microarray Analysis of the Hemagglutinins from Modern and Pandemic Influenza Viruses Reveals Different Receptor Specificities. *J. Mol. Biol.* **2006**, doi:10.1016/j.jmb.2005.11.002.

32. Jia, D.; Rahbar, R.; Chan, R.W.Y.; Lee, S.M.Y.; Chan, M.C.W.; Wang, B.X.; Baker, D.P.; Sun, B.; Malik Peiris, J.S.; Nicholls, J.M.; et al. Influenza Virus Non-Structural Protein 1 (NS1) Disrupts Interferon Signaling. *PLoS One* **2010**, doi:10.1371/journal.pone.0013927.

33. Westenius, V.; Mäkelä, S.M.; Julkunen, I.; Österlund, P. Highly Pathogenic H5N1 Influenza A Virus Spreads Efficiently in Human Primary Monocyte-Derived Macrophages and Dendritic Cells. *Front. Immunol.* **2018**, doi:10.3389/fimmu.2018.01664.

34. Liu, Q.; Yang, J.; Huang, X.; Liu, Y.; Han, K.; Zhao, D.; Zhang, L.; Li, Y. Transcriptomic Profile of Chicken Bone Marrow-Derive Dendritic Cells in Response to H9N2 Avian Influenza A Virus. *Vet. Immunol. Immunopathol.* **2020**, doi:10.1016/j.vetimm.2019.109992.

35. Dienz, O.; Rud, J.G.; Eaton, S.M.; Lanthier, P.A.; Burg, E.; Drew, A.; Bunn, J.; Suratt, B.T.; Haynes, L.; Rincon, M. Essential Role of IL-6 in Protection against H1N1 Influenza Virus by Promoting Neutrophil Survival in the Lung. *Mucosal Immunol.* **2012**, doi:10.1038/mi.2012.2.

36. Moriyama, M.; Ichinohe, T. High Ambient Temperature Dampens Adaptive Immune Responses to Influenza A Virus Infection. *Proc. Natl. Acad. Sci. U. S. A.* **2019**, doi:10.1073/pnas.1815029116.

37. Nüssing, S.; Sant, S.; Koutsakos, M.; Subbarao, K.; Nguyen, T.H.O.; Kedzierska, K. Innate and Adaptive T Cells in Influenza Disease. *Front. Med.* 2018.

38. Lee, L.Y.H.; Ha, D.L.A.; Simmons, C.; De Jong, M.D.; Chau, N.V.V.; Schumacher, R.; Yan, C.P.; McMichael, A.J.; Farrar, J.J.; Smith, G.L.; et al. Memory T Cells Established by Seasonal Human Influenza A Infection Cross-React with Avian Influenza A (H5N1) in Healthy Individuals. *J. Clin. Invest.* **2008**, doi:10.1172/JCI32460.

39. Wilkinson, T.M.; Li, C.K.F.; Chui, C.S.C.; Huang, A.K.Y.; Perkins, M.; Liebner, J.C.; Lambkin-Williams, R.; Gilbert, A.; Oxford, J.; Nicholas, B.; et al. Preexisting Influenza-Specific CD4 + T Cells Correlate with Disease Protection against Influenza Challenge in Humans. *Nat. Med.* **2012**, doi:10.1038/nm.2612.

40. Loh, L.; Wang, Z.; Sant, S.; Koutsakos, M.; Jegaskanda, S.; Corbett, A.J.; Liu, L.; Fairlie, D.P.; Crowe, J.; Rossjohn, J.; et al. Human Mucosal-Associated Invariant T Cells Contribute to Antiviral Influenza Immunity via IL-18-Dependent Activation. *Proc. Natl. Acad. Sci. U. S. A.* **2016**, doi:10.1073/pnas.1610750113.

41. Reemers, S.S.; van Leenen, D.; Groot Koerkamp, M.J.; van Haarlem, D.; van de Haar, P.; van Eden, W.; Vervelde, L. Early Host Responses to Avian Influenza A Virus Are Prolonged and Enhanced at Transcriptional Level Depending on Maturation of the Immune System. *Mol. Immunol.* **2010**, doi:10.1016/j.molimm.2010.03.008.

42. Pantin-Jackwood, M.J.; Smith, D.M.; Wasilenko, J.L.; Cagle, C.; Shepherd, E.; Sarmento, L.; Kapczynski, D.R.; Afonso, C.L. Effect of Age on the Pathogenesis and Innate Immune Responses in Pekin Ducks Infected with Different H5N1 Highly Pathogenic Avian Influenza Viruses. *Virus Res.* **2012**, doi:10.1016/j.virusres.2012.04.015.

43. Ranaware, P.B.; Mishra, A.; Vijayakumar, P.; Gandhale, P.N.; Kumar, H.; Kulkarni, D.D.; Raut, A.A. Genome Wide Host Gene Expression Analysis in Chicken Lungs Infected with Avian Influenza Viruses. *PLoS One* **2016**, doi:10.1371/journal.pone.0153671.

44. Xing, Z.; Cardona, C.J.; Li, J.; Dao, N.; Tran, T.; Andrada, J. Modulation of the Immune Responses in Chickens by Low-Pathogenicity Avian Influenza Virus H9N2. *J. Gen. Virol.* **2008**, doi:10.1099/vir.0.83362-0.

45. Nang, N.; Lee, J.; Song, B.; Kang, Y.; Kim, H.; Seo, S. Induction of Inflammatory Cytokines and Toll-like Receptors in Chickens Infected with Avian H9N2 Influenza Virus. *Vet. Res.* **2011**, doi:10.1186/1297-9716-42-64.

46. Jiang, H.; Yu, K.; Kapczynski, D.R. Transcription Factor Regulation and Cytokine Expression Following in Vitro Infection of Primary Chicken Cell Culture with Low Pathogenic Avian Influenza Virus. *Virol. J.* **2013**, doi:10.1186/1743-422X-10-342.

47. Xing, Z.; Cardona, C.J.; Anunciacion, J.; Adams, S.; Dao, N. Roles of the ERK MAPK in the Regulation of Proinflammatory and Apoptotic Responses in Chicken Macrophages Infected with H9N2 Avian Influenza Virus. *J. Gen. Virol.* **2010**, doi:10.1099/vir.0.015578-0.

48. Post, J.; Burt, D.W.; Cornelissen, J.B.W.J.; Broks, V.; Van Zoelen, D.; Peeters, B.; Rebel, J.M.J. Systemic Virus Distribution and Host Responses in Brain and Intestine of Chickens Infected with Low Pathogenic or High Pathogenic Avian Influenza Virus. *Virol. J.* **2012**, doi:10.1186/1743-422X-9-61.

49. Cui, Z.; Hu, J.; He, L.; Li, Q.; Gu, M.; Wang, X.; Hu, S.; Liu, H.; Liu, W.; Liu, X.; et al. Differential Immune Response of Mallard Duck Peripheral Blood Mononuclear Cells to Two Highly Pathogenic Avian Influenza H5N1 Viruses with Distinct Pathogenicity in Mallard Ducks. *Arch. Virol.* **2014**, doi:10.1007/s00705-013-1820-6.

50. Kumar, A.; Vijayakumar, P.; Gandhale, P.N.; Ranaware, P.B.; Kumar, H.; Kulkarni, D.D.; Raut, A.A.; Mishra, A. Genome-Wide Gene Expression Pattern Underlying Differential Host Response to High or Low Pathogenic H5N1 Avian Influenza Virus in Ducks. *Acta Virol.* **2017**, doi:10.4149/av_2017_01_66.

51. Barber, M.R.W.; Aldridge, J.R.; Webster, R.G.; Magor, K.E. Association of RIG-I with Innate Immunity of Ducks to Influenza. *Proc. Natl. Acad. Sci. U. S. A.* **2010**, doi:10.1073/pnas.1001755107.

52. Jansen, C.A.; De Geus, E.D.; Van Haarlem, D.A.; Van De Haar, P.M.; Löndt, B.Z.; Graham, S.P.; Göbel, T.W.; Van Eden, W.; Brookes, S.M.; Vervelde, L. Differential Lung NK Cell Responses in Avian Influenza Virus Infected Chickens Correlate with Pathogenicity. *Sci. Rep.* **2013**, doi:10.1038/srep02478.

53. Avian Influenza A (H5N1) Infection in Humans. *N. Engl. J. Med.* **2005**,

doi:10.1056/nejmra052211.

54. de Vries, R.D.; Herfst, S.; Richard, M. Avian Influenza A Virus Pandemic Preparedness and Vaccine Development. *Vaccines* 2018.

55. Coughlan, L. Factors Which Contribute to the Immunogenicity of Non-Replicating Adenoviral Vectored Vaccines. *Front. Immunol.* 2020.

56. Wang, Y.; Deng, L.; Kang, S.M.; Wang, B.Z. Universal Influenza Vaccines: From Viruses to Nanoparticles. *Expert Rev. Vaccines* 2018.

57. Krammer, F. The Human Antibody Response to Influenza A Virus Infection and Vaccination. *Nat. Rev. Immunol.* 2019.

58. de Vries, R.D.; Rimmelzwaan, G.F. Viral Vector-Based Influenza Vaccines. *Hum. Vaccines Immunother.* 2016.

59. Benjamin, E.; Wang, W.; McAuliffe, J.M.; Palmer-Hill, F.J.; Kallewaard, N.L.; Chen, Z.; Suzich, J.A.; Blair, W.S.; Jin, H.; Zhu, Q. A Broadly Neutralizing Human Monoclonal Antibody Directed against a Novel Conserved Epitope on the Influenza Virus H3 Hemagglutinin Globular Head. *J. Virol.* **2014**, doi:10.1128/jvi.03562-13.

60. Raymond, D.D.; Bajic, G.; Ferdman, J.; Suphaphiphat, P.; Settembre, E.C.; Moody, M.A.; Schmidt, A.G.; Harrison, S.C. Conserved Epitope on Influenza-Virus Hemagglutinin Head Defined by a Vaccine-Induced Antibody. *Proc. Natl. Acad. Sci. U. S. A.* **2018**, doi:10.1073/pnas.1715471115.

61. Nolan, T.M.; Richmond, P.C.; Skeljo, M. V.; Pearce, G.; Hartel, G.; Formica, N.T.; Höschler, K.; Bennet, J.; Ryan, D.; Papanaoum, K.; et al. Phase I and II Randomised Trials of the Safety and Immunogenicity of a Prototype Adjuvanted Inactivated Split-Virus Influenza A (H5N1) Vaccine in Healthy Adults. *Vaccine* **2008**, doi:10.1016/j.vaccine.2008.05.077.

62. Ehrlich, H.J.; Müller, M.; Oh, H.M.L.; Tambyah, P.A.; Joukhadar, C.; Montomoli, E.; Fisher, D.; Berezuk, G.; Fritsch, S.; Löw-Baselli, A.; et al. A Clinical Trial of a Whole-Virus H5N1 Vaccine Derived from Cell Culture. *N. Engl. J. Med.* **2008**, doi:10.1056/nejmoa073121.

63. Beran, J.; Abdel-Messih, I.A.; Raupachova, J.; Hobzova, L.; Fragapane, E. A Phase III, Randomized, Open-Label Study to Assess the Tolerability and Immunogenicity of an H5N1 Influenza Vaccine Administered to Healthy Adults with a 1-, 2-, 3-, or 6-Week Interval between First and Second Doses. *Clin. Ther.* **2010**, doi:10.1016/S0149-2918(11)00024-5.

64. Couch, R.B.; Patel, S.M.; Wade-Bowers, C.L.; Niño, D. A Randomized Clinical Trial of an Inactivated Avian Influenza A (H7N7) Vaccine. *PLoS One* **2012**, doi:10.1371/journal.pone.0049704.

65. Matsuda, K.; Huang, J.; Zhou, T.; Sheng, Z.; Kang, B.H.; Ishida, E.; Griesman, T.; Stuccio, S.; Bolkhovitinov, L.; Wohlbold, T.J.; et al. Prolonged Evolution of the Memory B Cell Response Induced by a Replicating Adenovirus-Influenza H5 Vaccine. *Sci. Immunol.* **2019**, doi:10.1126/sciimmunol.aau2710.

66. Duong, T.N.; Thiem, V.D.; Anh, D.D.; Cuong, N.P.; Thang, T.C.; Huong, V.M.;

Chien, V.C.; Phuong, N.T.L.; Montomoli, E.; Holt, R.; et al. A Phase 2/3 Double Blinded, Randomized, Placebo-Controlled Study in Healthy Adult Participants in Vietnam to Examine the Safety and Immunogenicity of an Inactivated Whole Virion, Alum Adjuvanted, A(H5N1) Influenza Vaccine (IVACFLU-A/H5N1). *Vaccine* **2020**, doi:10.1016/j.vaccine.2019.11.059.
